# Enhancing Maturation of Human Neuromuscular Organoids via Electrical Stimulation

**DOI:** 10.1002/advs.202522762

**Published:** 2026-06-22

**Authors:** Chrysanthi‐Maria Moysidou, Inês Afonso Martins, Ismail Amr El‐Shimy, Iacopo Bicci, Donatella Cea, Christina Bukas, Isra Mekki, Mara‐Camelia Rusu, Aylin Nebol, Ines Lahmann, Marie Piraud, Enrico Klotzsch, Mina Gouti

**Affiliations:** ^1^ Max Delbrück Center for Molecular Medicine (MDC) Berlin Germany; ^2^ Helmholtz AI, Computational Health Center (CHC) Helmholtz Munich Neuherberg Germany; ^3^ Berlin Institute of Health Center for Regenerative Therapies (BCRT) Berlin Germany; ^4^ Berlin Institute of Health Experimental and Clinical Research Center (ECRC) Berlin Germany; ^5^ Charité Universitätsmedizin Berlin Germany; ^6^ Department of Pediatric Neurology Charité Universitätsmedizin Berlin Berlin Germany

**Keywords:** bioengineering, biophysical cues, electrical stimulation, EPS‐NMOs, neuromuscular organoids, neuromuscular systems, organoid maturation

## Abstract

Organoids derived from human pluripotent stem cells (hPSCs) are emerging as powerful models for studying development and disease. Despite their physiological relevance, the predictive power of organoids remains limited by the immature state of the constituent cells, posing a major challenge for mechanistic studies of adult physiology and late‐onset disorders. Here, we establish a strategy for enhancing the maturation status of human neuromuscular organoids (NMOs) through chronic Electrical Pulse Stimulation (EPS). We demonstrate that low‐frequency EPS, applied during the early stages of NMO development and maintained over several weeks, enhances neuromuscular maturation and functional output. Independent of stimulation waveform dynamics, EPS‐trained NMOs (EPS‐NMOs) display stronger and more frequent spontaneous contractions that persist long after stimulation has ceased. Quantitative imaging and transcriptomic analyses reveal a robust improvement in EPS‐NMO skeletal muscle and neural tissue morphology, coordinated regulation of lineage‐specific biomarkers, and upregulation of gene programs associated with neuromuscular maturation. Mechanobiological measurements further demonstrate increased tissue stiffness and faster relaxation dynamics in EPS‐NMOs, consistent with enhanced excitation‐contraction coupling (ECC) and force generation. Collectively, these findings establish EPS as a powerful, non‐invasive, and on‐demand modality for promoting the morphological and functional maturation of complex organoid systems.

## Graphical abstract

A framework for on‐demand and non‐invasive exposure of human neuromuscular organoids (NMOs) to electrical stimuli is established to promote their maturation. The robustness and effectiveness of different stimulation regimes are evaluated via thorough characterization of organoid tissue structure and contraction capacity. Chronic electrical stimulation, applied from early organoid growth stages, leads to neuromuscular tissues of more mature morphological and functional profiles.

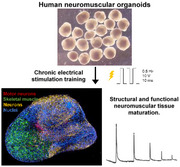

## 1 Introduction

Advances in stem cell technology have enabled the generation of three‐dimensional (3D) self‐organizing cellular ensembles, namely organoids [1, 2]. Unlike two‐dimensional (2D) cell culture models, human organoids capture the 3D cytoarchitecture, cellular diversity, and intercellular interactions of their in vivo counterparts, thus providing powerful in vitro systems to study human development and disease [1, 3]. As such, organoids are considered paradigm‐shifting tools in biomedical research and are increasingly employed in modeling various human tissues, including intestine [4], liver [5, 6], lung [7, 8], heart [9], and brain [10, 11], facilitating in vitro studies of organogenesis and disease at an unprecedented level [1, 12].

Despite these advances, achieving adult‐like tissue maturation states in organoids remains a challenge [2, 13, 14]. Attaining such levels of maturation is critical for employing organoids in adult‐onset disease modeling (e.g., Amyotrophic Lateral Sclerosis (ALS), Alzheimer's disease (AS), Age‐related Macular Degeneration (AMD), cancer), as well as in drug discovery pipelines and personalized medicine applications [2, 15].

Several strategies have been developed to promote organoid maturation, including long‐term culture to approximate developmental timelines [3], co‐culture or fusion with other cell types and/or organoids [16, 17, 18], and transplantation into host tissues [19, 20, 21, 22]. Despite the valuable insight these approaches offer, organoids remain largely constrained to fetal or early post‐natal stages [2, 12, 23, 24, 25], indicating that essential cues are missing. Recent work has made strides in identifying such factors, highlighting the differential effect of distinct stimuli on tissue‐specific maturation pathways [13, 17]. Among the most promising approaches are complex media formulations, hormonal stimulation [13, 26, 27], and integration of biophysical cues [2, 14, 28, 29], such as mechanical loading and electrical stimulation, which has emerged as one of the most potent drivers for functional tissue maturation [30, 31, 32].

Bioelectric manipulation of tissues can be traced back to Luigi Galvani's experiments on frog leg muscle twitches, demonstrating that muscle contraction is driven by electrical stimuli, laying the foundation of modern electrophysiology [33]. We now know that the fundamental mechanism by which motor neuron action potentials are converted into muscle fiber mechanical contraction is excitation‐contraction coupling (ECC) [34, 35, 36]. In skeletal muscle, ECC starts at the neuromuscular junction (NMJ) upon arrival of motor neuron action potentials, triggering the release of acetylcholine (ACh) from the presynaptic terminal into the synaptic cleft. Binding of ACh to its receptors at the post‐synaptic terminal induces depolarization of the sarcolemma, which is propagated down the muscle fibers. Such rapid changes in the muscle membrane electrical properties cause the release of large amounts of Ca^2+^ into the sarcoplasm and structural conformation of myosin and actin proteins that slide along each other, leading to myofibril contractions [34, 36, 37]. Electrical Pulse Stimulation (EPS) is commonly used to study and modulate ECC in in vitro models, emulating motor neuron activation of skeletal muscle cultures [38, 39, 40, 41, 42, 43, 44, 45]. While EPS has been widely applied to 2D myotube cultures to enhance contractility and metabolic function, it is typically introduced only at late differentiation states and in the absence of motor neuron input [46, 47]. As a result, most studies do not address how bioelectrical cues during early developmental stages might influence reciprocal neuron‐muscle signaling, self‐organization, and NMJ formation and maturation.

Human neuromuscular organoids (NMOs) offer a powerful platform to overcome these limitations. We have previously demonstrated that self‐organizing human NMOs closely capture the developmental trajectory, tissue organization, and complexity of their in vivo counterpart, including the formation of morphologically and functionally competent NMJs that drive spontaneous contractions [48]. Here, we hypothesized that introducing bioelectrical cues through controlled EPS training could further promote NMO maturation, acting as a physiological stimulus that is normally absent. To test this hypothesis, we developed a framework for low‐frequency EPS training of NMOs and provided proof‐of‐concept that electrical stimulation can accelerate and enhance their functional maturation. Using acute stimulation assays and NMJ functionality as a readout, we first identified early NMO developmental stages as the optimum time window to initiate EPS training. While acute/short‐term stimulation induced improvements in NMJ activity, these effects were transient, suggesting that prolonged stimulation is required to achieve stable and long‐term maturation. We therefore established chronic EPS protocols, applying stable or dynamic pacing for approximately one month. Regardless of pacing parameters, chronic EPS elicited a significant boost in NMO maturation status, as evidenced by a marked increase in NMJ absolute number, size, and innervation levels, which translated into more frequent and stronger spontaneous contractions of EPS‐NMOs. Strikingly, such enhanced activity persists for several days after training stops. Transcriptomic analysis revealed that chronic EPS, under stable or increasing pulse parameters, induced a significant upregulation of several genes involved in muscle contraction, myelination, and neuronal functions, consistent with accelerated neuromuscular maturation. Quantitative image analysis further substantiated these findings, revealing tissue‐specific EPS‐driven maturation effects. EPS‐NMOs contained a significantly higher number of glial cells, compared to non‐paced control NMOs, with improved localization and projection of motor neurons in the muscle region, which, in turn, exhibited significantly higher numbers of more elongated and better aligned myofibers. Importantly, mechanobiological characterization assays indicated that EPS‐NMOs acquired significantly stiffer tissue properties and faster relaxation dynamics compared to non‐paced control NMOs, consistent with enhanced ECC and contractile output. Overall, our findings establish chronic low‐frequency EPS as a robust, non‐invasive, and tunable approach to promote NMO maturation. Our study not only showcases the benefits of integrating bioelectrical cues with organoid models but it also highlights the importance of introducing such biophysical cues from early developmental stages, providing a flexible framework for guided, on‐demand maturation of complex organoid models.

## 2 Results

### 2.1 Establishing an EPS Training Paradigm in NMOs

**FIGURE 1:**
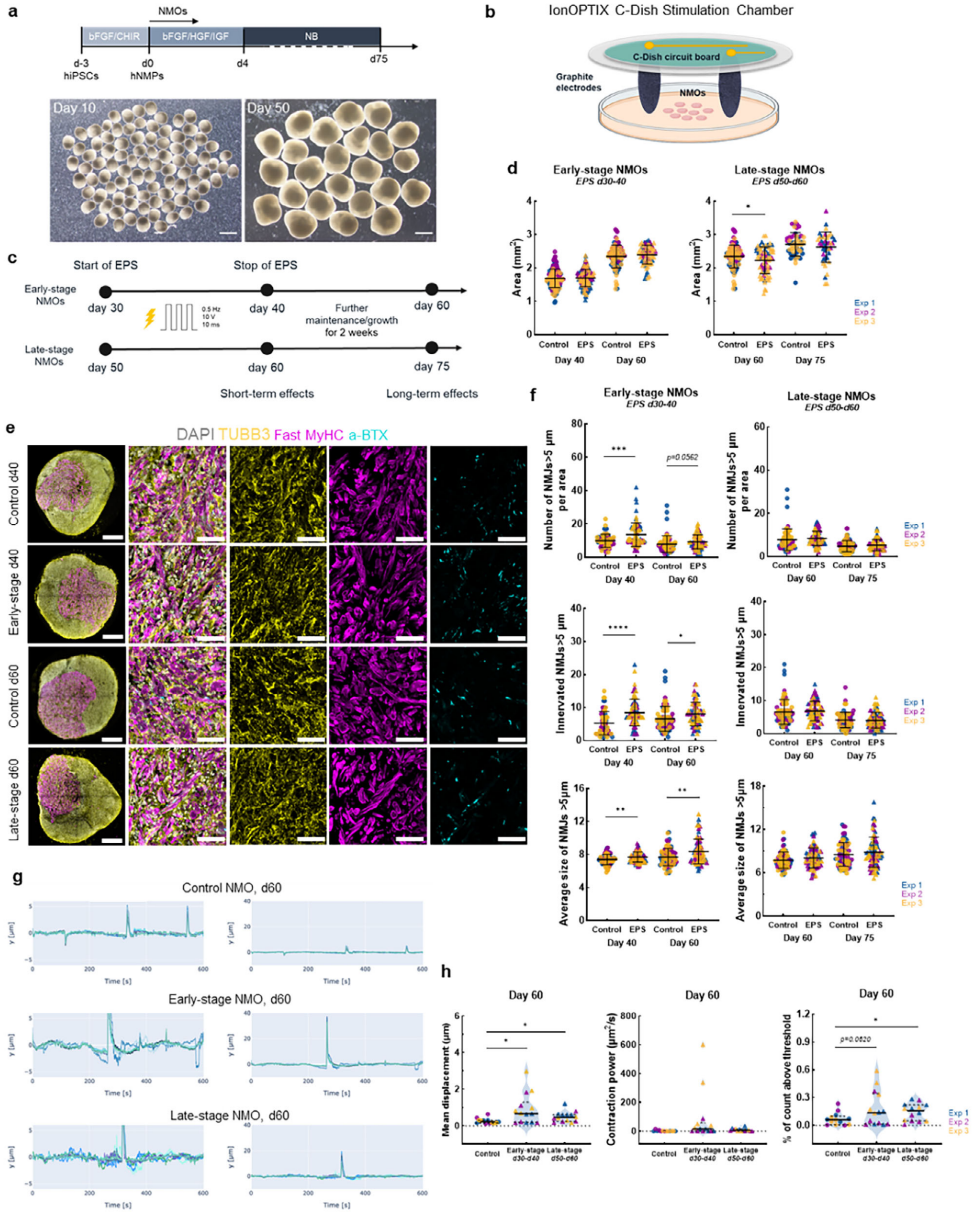
Defining the developmental window during which NMOs respond to EPS training. (a) Schematic representation of the NMO generation from hiPSC‐derived NMPs. Representative brightfield images of day 10 and day 50 WTC^mTTNGFP^ NMOs. Scale bar 1 mm. (b) Schematic illustration of the EPS training experimental setup, depicting one well of the IonOptix stimulation chamber, with NMOs between a set of C‐Dish graphite electrodes. (c) Experimental design for the identification of the NMO developmental stage at which EPS training can start. Day 30 or 50 WTC^mTTNGFP^ NMOs are exposed to AC stimulation with rectangular electrical bipolar pulses of 0.5 Hz, 10 V, 10 ms, for 30 min, every other day, for 10 days, after which NMOs are collected for short‐term EPS downstream analysis, while some are further maintained for long‐term EPS effect evaluation. (d) Plots illustrating the size of Early‐/Late‐stage and non‐paced WTC^mTTNGFP^ NMOs expressed as Area (mm^2^). The mean ± SD is shown for each experimental group (control in dots, EPS in triangle). Each datapoint represents one NMO (Early‐stage NMOs: day 40 control *n* = 191, EPS *n* = 141, day 60 control *n* = 98, EPS *n* = 69; Late‐stage NMOs: day 60 control *n* = 98, EPS *n* = 72). (e) Confocal images of immunofluorescently labeled sections of WTC^mTTNGFP^ NMOs for neuronal (tubulin‐β‐3; TUBB3, in yellow), muscle (Fast Myosin Heavy Chain; MyHC, in magenta) and NMJ biomarkers (a‐bungarotoxin; α‐ΒΤΧ in cyan), counterstained for DAPI (in grey). Representative whole NMO images are shown for each experimental group (scale bar: 250 µm), at each timepoint of interest, along with 63× fields of view of the respective NMOs (scale bar 50 µm). (f) Quantification of NMJ features based on 63× micrographs in e. Plots depict the absolute number per area (i.e., per 63× micrograph), innervation, and average size of NMJs>5 µm on day 40 and day 60 for Early‐stage NMOs and on day 60 and day 75 Late‐stage NMOs. The mean ± SD of *N = 3* independent experiments is shown for each experimental group. Each datapoint represents one 63× micrograph (control: day 40 *n* = 37 from 11 NMOs, day 60 *n* = 73 from 13 NMOs, day 75 *n* = 78 from 13 NMOs; Early‐stage NMOs, day 40 *n* = 78 from 13 NMOs, day 60 *n* = 79 from 13 NMOs; Late‐stage NMOs: day 60 *n* = 74 from 12 NMOs, day 75 *n* = 85 micrographs from 15 NMOs). (g) Representative *Time Series* plots and corresponding close‐up insets, illustrating the spontaneous contractile activity of WTC^mTTNGFP^ non‐paced control NMOs and EPS‐NMOs on day 60, during a 10‐min live recording. (h) Quantitative analysis of the spontaneous contractile activity of WTC^mTTNGFP^ non‐paced control NMOs and EPS‐NMOs on day 60, expressed as *Mean displacement* (µm)*, Contraction power* (µm^2^ s^−1^), and *% of count above threshold*. Each datapoint represents one NMO (i.e., one 10‐min live recording; control: *n* = 13 NMOs, Early‐stage: *n* = 14 NMOs, Late‐stage: *n* = 14 NMOs). In all plots, data from *N* = 3 independent experiments are analyzed by unpaired *t*‐test with Welch correction (^*^p ≤ 0.05; ^**^ *p* ≤ 0.01; ^***^ *p* ≤ 0.001; ^****^ *p* ≤ 0.0001).

We have previously established a robust protocol for the generation of NMOs in 3D from neuromesodermal progenitors (NMPs), derived from various human pluripotent stem cell (hPSC) lines [48]. A defining characteristic of 3D NMOs is the self‐organization of spinal cord neurons and skeletal muscle cells into functional neuromuscular junctions (NMJs). NMJs start forming around NMO developmental day 25–30, characteristic acetyl‐choline receptor (AChR) clusters can be clearly detected by day 30, and by day 50, functional NMJ‐like structures are formed, driving NMO spontaneous contraction [48]. Based on this protocol, we derived NMPs from the human induced pluripotent stem cells (hiPSCs) KOLF2.1J [49] (i.e., KOLF) and WTC‐11, fluorescently tagged with mEGFP to target titin (TTN) protein (i.e., WTC^mTTNGFP^) [50] and, subsequently, generated NMOs (Figure 1a).

We then used these NMOs to explore the potential of EPS training in enhancing the maturation status and functionality of organoids. As NMJs are established and maturing between days 30–50, we hypothesized that the timing of EPS would influence how NMOs respond to electrical activity. We therefore compared two developmental windows of NMOs: (i) Early‐stage NMOs, day 30, corresponding to the onset of NMJ formation and (ii) Late‐stage NMOs, day 50, corresponding to the stage when functional NMJs are already established. To deliver EPS, we used a multi‐channel pacing system, compatible with standard culture formats (Figure 1b), previously used for chronic electrical stimulation of cardiomyocyte cultures [31, 51, 52, 53, 54] and for modeling exercise in vitro [38, 41, 42, 55, 56, 57]. Low‐frequency, low‐voltage stimulation has been previously used to promote skeletal muscle maturation under physiological conditions [58]. Low‐frequency stimulation emulates motor neuron basal firing rate and allows for physiological contraction‐relaxation cycles, enabling muscle fibers to fully relax before the next cycle, and preventing tetanic contractions. Low‐voltage stimuli are used to balance such physiological relevance with cell/tissue viability, as higher voltages may induce electrochemical reaction byproducts and/or electrode overheating, which can compromise cells and tissues [58, 59]. Based on these principles, we screened a range of stimulation pulse parameters, including frequency (0.2–3 Hz), pulse width (2‐10 ms), and voltage (2–30 V) to identify conditions that elicited robust, synchronous, physiological contractions on day 65 NMOs. Rectangular biphasic AC electrical pulses of 0.5 Hz, 10 V, and 10 ms reliably induce a synchronous NMO contractile response, followed by full relaxation between pulses (Movies S1 and S2). These findings support the use of EPS‐training under physiologically relevant conditions to stimulate NMOs and induce synchronous muscle contractions.

### 2.2 Electrical Pulse Stimulation Modulates NMJ Morphology and Function in a Time‐Dependent Manner

Using these optimized parameters, we subjected WTC^mTTNGFP^ NMOs to acute/short training of 30 min of EPS (0.5 Hz, 10 V, 10 ms) every other day for 10 days (Figure 1c). Early‐stage NMOs were trained from day 30 to day 40 and analyzed on day 40 and on day 60, and Late‐stage NMOs were trained from day 50 to day 60 and analyzed on day 60 and on day 75 (Movies S3–S7), in order to determine the short‐ and long‐term effects of EPS on NMO morphology and function across developmental stages.

First, we compared the size of EPS‐NMOs with non‐paced control NMOs. EPS training did not interfere with the growth of Early‐stage NMOs (EPS between d30‐d40), which followed the same growth pattern as non‐paced control NMOs (Figure 1d and Figure S1a,c), exhibiting a normal increase in size on day 40 and on day 60. In contrast, EPS training on Late‐stage NMOs (d50‐60) induced a transient reduction in NMO size at day 60, but this effect resolved by day 75, when trained and control NMOs had comparable sizes. (Figure 1d and Figure S1b,c). In addition, to examine whether EPS‐training affected cell viability, we assessed apoptosis in NMOs using cleaved Caspase‐3 (cCas‐3) immunofluorescence analysis (Figure S2a). Quantification of cCas‐3 expression in the neural and muscle compartments showed no difference in the expected baseline apoptotic levels between control and EPS‐NMOs, indicating that EPS‐training did not affect NMO survival (Figure S2b). This was also confirmed by quantification of TUNEL^+^ cell expression, as a late‐stage marker of cells undergoing apoptosis, which revealed no significant differences between control and EPS‐NMOs.

Next, we examined the effects of electrical stimulation on NMJ development and morphology by analyzing the NMJ number, size, and innervation in Early‐stage and Late‐stage EPS‐NMOs compared to non‐paced control NMOs (Figure 1e). In this context, α‐BTX^+^ AChR clusters are used as a proxy for postsynaptic NMJ structures, while innervated NMJs are defined by co‐localization with neuronal markers and will be referred to hereafter as NMJ‐like structures. These were evaluated at two timepoints; on the day EPS‐training stops (day 40, day 60) and ∼2 to 3 weeks post‐EPS training (day 60, day 75), in order to assess both short‐ and long‐term effects, respectively. Early‐stage EPS produced a consistent increase in NMJ number and innervation at day 40, confirmed across three independent differentiations (Figure 1f and Figure S3). Although this effect persisted post‐EPS for both NMJs > 2 µm and larger NMJs > 5 µm, it was not as significant on day 60. Moreover, NMJ‐like structure morphological analysis revealed that NMJs > 5 µm were significantly larger in Early‐stage EPS‐NMOs compared to controls at both day 40 and day 60, indicating a lasting positive effect on NMJ maturation. In contrast, Late‐stage EPS‐training did not induce any significant changes in the size and innervation of NMJ‐like structures compared to non‐paced control NMOs at both timepoints (Figure 1f and Figure S3).

To further investigate the effects of EPS‐training on NMJ status, we shifted our focus to the functional output of NMOs, analyzing their spontaneous contractile activity on day 60; a developmental stage associated with more mature and functional NMJ‐like structures that support skeletal muscle contraction in NMOs. Representative *Time Series* plots revealed enhanced contractility in both EPS‐trained groups, which exhibited contractions of larger displacement (i.e., *y‐*axis; maximum distance from the organoid border, corresponding to 0 µm), compared to non‐paced control NMOs (Figure 1g and Movies S8–S10). This was especially pronounced in Early‐stage NMOs, despite EPS having ended ∼3 weeks earlier, suggesting long‐lasting functional benefits. Quantitative analysis of contractile features confirmed these observations, revealing an EPS‐induced increase in the *Mean Displacement* (i.e., the average absolute displacement of the NMO from its border, corresponding to 0 µm, for the duration of the recording), *Contraction power* (i.e., the area under the curve of the squared time series over the duration of the recording, reflecting the overall magnitude of the signal), as well as a boost in the number of contractions above threshold in both experimental groups, with a significant increase in Late‐stage EPS‐NMOs (Figure 1h). The trend toward higher contractile output of Early‐stage NMOs on day 60 correlated with their significantly larger NMJ‐like structures, whereas Late‐stage NMOs exhibited no further increase in NMJ‐like structure size, suggesting that different mechanisms may underlie these responses. EPS training during the early NMO developmental window (d30‐d40) coincides with NMJ formation and precedes the onset of spontaneous contractile activity (∼d50). In contrast, Late‐stage NMOs have already reached such milestones when EPS is applied (d50‐d60) and can respond independently of NMJ function (Movies S6 and S7). In line with this, pharmacological blockade of NMJ‐like structures using curare (10 µM) did not abolish EPS‐induced contractions in day 60 NMOs, which continue to exhibit robust responses to electrical pulses (1 Hz, 10 V,10 ms) (Movie S11). This indicates that EPS can directly induce muscle contractions independently of synaptic transmission. We also note that spontaneous activity may occur alongside EPS induced contractions, which can be observed as variability/asynchronous NMO contractions to the pulse frequency in some recordings (i.e., one or more NMOs may also contract spontaneously, such as in Movie S1)

Together, our results demonstrate that EPS training effectively modulates NMJ morphology and enhances overall neuromuscular functionality even after relatively short‐term training (i.e., 10 days). However, the timing of training proved decisive for neuromuscular maturation, with early EPS‐training coinciding with NMJ formation, yielding the most robust and enduring outcomes.

### 2.3 Chronic Electrical Pulse Stimulation Enhances NMJ Maturation Status, Independent of Pulse Parameters

Having identified early developmental stages as the optimal developmental window for effective NMO EPS‐training, we then investigated how the duration of stimulation and the modulation of pulse parameters influence NMO maturation.

**FIGURE 2:**
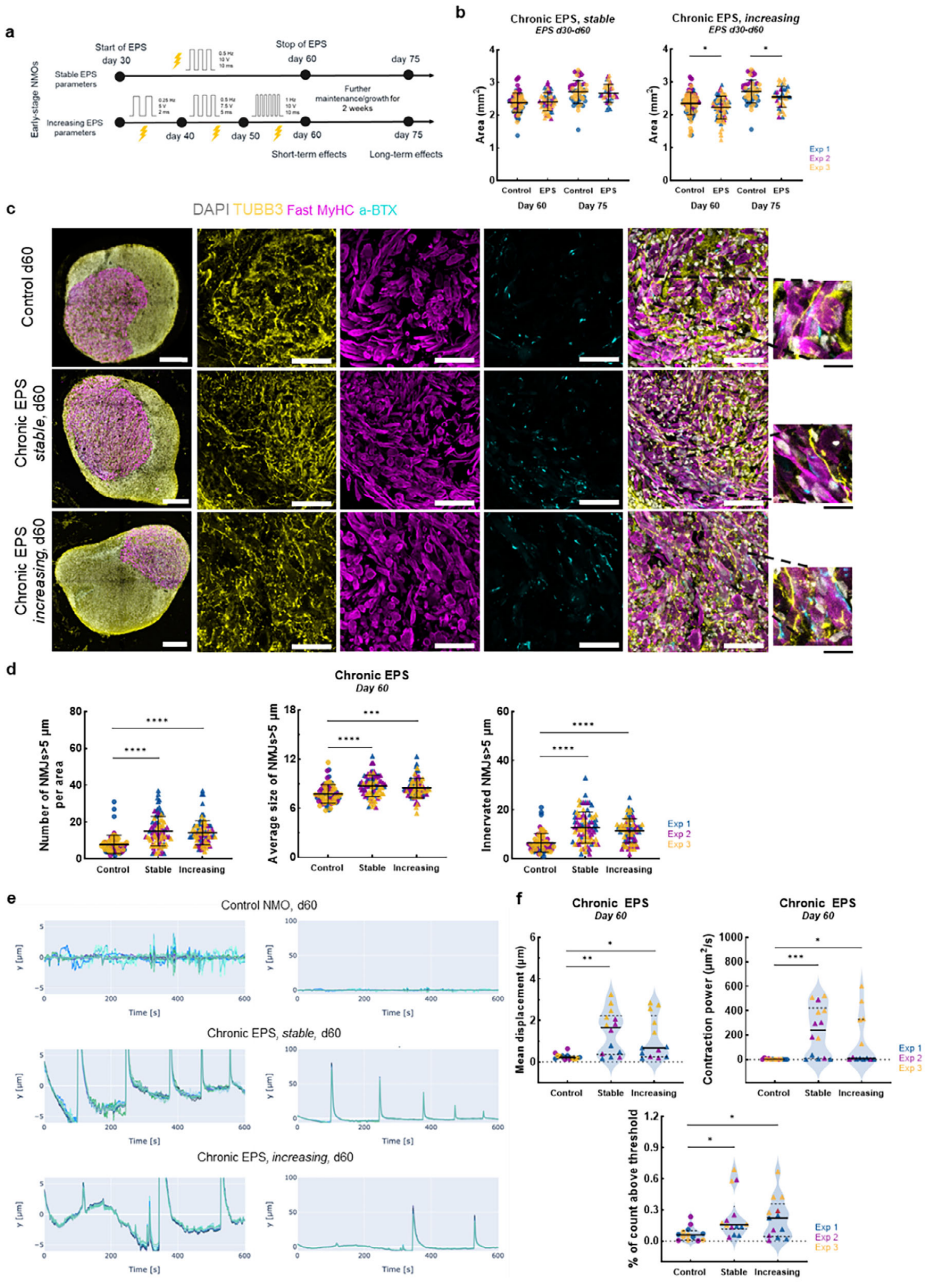
Modulation of NMJ function via chronic EPS training of Early‐stage NMOs. (a) Schematic illustration of the modified experimental design for chronic EPS‐training of NMOs. Early‐stage (day 30) NMOs were subjected to EPS assays of either stable or increasing parameters, every other day, for ∼one month (day 60). NMOs were analyzed on day 60 (short‐term) and on day 75 (long‐term) to evaluate the effects of chronic EPS on NMJ morphology and function. (b) Plots illustrating the size of WTC^mTTNGFP^ non‐paced control NMOs and EPS‐NMOs (expressed as Area (mm^2^)). Each datapoint represents one NMO (control: day 60 n = 86, day 75 *n* = 51 NMOs; Chronic EPS, stable: day 60 *n* = 57, day 75 *n* = 54 NMOs; Chronic EPS, increasing: day 60 *n* = 72, day 75 *n* = 38 NMOs). (c) Confocal images of immunofluorescently labeled sections of WTC^mTTNGFP^ NMOs for neuronal (tubulin‐β‐3;TUBB3, in yellow), muscle (Fast Myosin Heavy Chain; MyHC, in magenta), and NMJ biomarkers (a‐bungarotoxin; α‐ΒΤΧ in cyan), counterstained for DAPI (in grey). Representative whole NMO images are shown for each experimental group, on day 60 (scale bar: 250 µm), along with 63× micrographs of the respective NMOs (scale bar 50 µm). Panels on the right correspond to the annotated areas (black dashed lines) in each 63× rendering, offering a close‐up look at the colocalization of NMJ, neural, and muscle markers (scale bar 20 µm). (d) Quantification of day 60 NMJs > 5 µm features, based on 63× micrographs in c. Plots show the absolute number per area (i.e., per 63× micrograph), innervation, and average size of NMJs > 5 µm on day 60 for WTC^mTTNGFP^ non‐paced control NMOs and EPS‐NMOs, trained under stable or increasing pulse parameters. The mean ± SD of *N = 3* independent experiments is shown for each experimental group. Each datapoint represents one 63× micrograph (control: *n* = 73 from 13 NMOs, Chronic EPS: stable: *n* = 84 from 14 NMOs; increasing: *n* = 84 from 14 NMOs). (e) Representative *Time Series* plots and corresponding close‐ups, illustrating the spontaneous contractile activity of WTC^mTTNGFP^ non‐paced control and EPS‐NMOs chronically trained on day 60, during a 10‐min live recording. (f) Quantitative analysis of the spontaneous contractile activity on day 60 WTC^mTTNGFP^ NMOs, expressed as *Mean displacement* µm)*, Contraction power* (µm^2^ s^−1^) plot and *% of count above threshold*. Each datapoint represents one NMO (i.e., one 10‐min live recording; control: *n* = 13 NMOs, Chronic EPS, stable: *n* = 14 NMOs, Chronic EPS, increasing: *n* = 13 NMOs). In all plots, data from *N* = 3 independent experiments are analyzed by unpaired *t*‐test with Welch correction (^*^ *p* ≤ 0.05; ^**^ *p* ≤ 0.01; ^***^ *p* ≤ 0.001; ^****^ *p* ≤ 0.0001).

To this end, Early‐stage NMOs (day 30) underwent 1 month of chronic EPS training under i) stable parameters (i.e., Chronic EPS, *stable*: 0.5 Hz, 10 V, 10 ms) or ii) progressively increasing parameters every 10 days (i.e., Chronic EPS, *increasing*: 0.5–1 Hz, 5–10 V, 2–10 ms), designed to follow the developmental trajectory of NMOs (Figure 2a). During this period, NMOs were subjected to EPS training for 30 mins, every other day, followed by analysis on day 60 (end of training) and on day 75 (two weeks post‐training) to determine short‐ and long‐term EPS effects, as before.

To assess if chronic EPS would elicit any changes in NMO growth trajectory, we quantified NMO size. NMOs exposed to stable stimulation parameters grew similarly to non‐paced control NMOs. In contrast, those subjected to increasing parameters were significantly smaller at both day 60 (when the EPS assay stops) and day 75 (two weeks post EPS; Figure 2b and Figure S4a,b). EPS‐NMOs continued to grow from day 60 to day 75, despite their reduced size, indicating preserved viability. cCas‐3 quantification showed no increase in apoptosis in EPS trained NMOs (Figure S5a,b). These findings suggest that increasing EPS‐training parameters shift the balance from growth toward accelerated differentiation and maturation, without evidence of overt apoptosis or widespread tissue damage. Similar findings were observed in quantitative analysis of TUNEL^+^ cells (Figure S5c,d).

Next, we examined the effects of chronic EPS on NMJ morphology and function. Transmission Electron Microscopy revealed neuron‐muscle contact sites (i.e., synaptic clefts) with ultra‐structures characteristic of NMJ formation, across control and EPS‐NMOs. These included synaptic vesicles at the presynaptic terminal of neurites, in close apposition to the muscle cell membrane, which in turn exhibits post‐synaptic densities, presence of basal lamina, and secondary synaptic cleft formation (Figure S6). Immunofluorescence analysis further confirmed the expression of presynaptic marker SV2 at axon terminals in close apposition, or co‐localized, with α‐BTX^+^ AChR clusters, both in non‐paced controls and in EPS‐NMOs (Figure S7a,b). We then used the same immunofluorescence image analysis pipeline as before to quantify NMJ‐like structure morphological features across three independent differentiations. Strikingly, one month of EPS‐training, applied under stable or increasing parameters, induced a highly significant increase in the absolute number, size, and innervation of mature NMJ‐like structures (>5 µm) on day 60 compared to non‐paced control NMOs (Figure 2c,d). However, this effect did not persist post‐EPS on day 75 (Figure S8a,b). In contrast, intermediate NMJ‐like structures (>2 µm) showed a sustained increase in number, size, and innervation in both EPS‐NMO groups, with a stronger effect on innervation for both training parameters on day 60. However, on day 75, this enhanced innervation persisted only in the increasing parameter NMO group, whereas the significantly higher numbers of intermediate NMJ‐like structures (>2 µm) persisted only in the stable parameter NMO group (Figure S8c).

Functional assays revealed that these morphological gains translated into improved contractile performance. Representative *Time Series* plots in Figure 2e clearly demonstrate a marked boost in NMO contractile activity upon 1‐month EPS training, with both EPS‐NMO groups exhibiting considerably larger displacement, compared to non‐paced control NMOs, as well as more contractions, especially in the Chronic EPS, *stable* NMO group (Movies s12–S14). Quantitative analysis further supports this, revealing significantly higher *Mean Displacement* (µm)*, Contraction power* (µm^2^ s^−1^), and contraction *counts above threshold* in both EPS‐NMO groups (Figure 2f). Importantly, such enhanced contractile function in EPS‐NMOs was sustained two weeks post‐training (Figure S9a,b and Movies S15–S17), highlighting the robust and persistent functional effects of such physical conditioning.

Notably, one‐month EPS training under stable parameters (i.e., Chronic EPS, *stable*: 0.5 Hz, 10 V, 10 ms, for 30 min, every other day) elicited the same benefit to the maturation status in an independent genetic background of KOLF NMOs. Both non‐paced control NMOs and EPS‐NMOs followed the same growth trajectory and comparable tissue segregation (Figure S10a,b), but EPS‐NMOs contained significantly higher numbers of intermediate NMJ‐like structures (>2 µm) and more mature NMJ‐like structures (>5 µm), of significantly larger size, and markedly increased levels of innervation (Figure S10c,d). These findings also explain the enhanced spontaneous contractile activity observed in KOLF EPS‐NMOs, exhibiting significantly higher *Contraction power* (µm^2^ s^−1^) and % of *counts above threshold* compared to non‐paced control NMOs (Figure S10e,f and Movies S18 and S19).

Taken together, our findings demonstrate that chronic EPS enhances NMJ maturation and functional neuromuscular outputs, without compromising normal NMO growth, tissue organisation, or cell viability. Importantly, such outcomes were achieved with both stable and progressive pacing, underlining the effectiveness of EPS in driving neuromuscular maturation.

### 2.4 Transcriptional Analysis Reveals Chronic Electrical Pulse Stimulation‐Induced Maturation of NMOs

External electrical stimulation has been shown to induce significant transcriptomic responses in neural and muscle tissue, associated with neuronal and synaptic function and motor activity [60], and with muscle structure, myogenesis, and contraction [61, 62, 63], among others.

To explore EPS effects on NMO transcriptomic profile, we performed bulk RNA sequencing on EPS‐NMOs and non‐paced control NMOs at two timepoints: on day 40, corresponding to Early‐stage, acute EPS‐training, and on day 60, corresponding to chronic NMO pacing, under stable and under increasing pulse parameters. Differential gene expression analysis of day 40 revealed significant upregulation of only four genes and significant downregulation of another four genes, indicating that short‐term, low‐frequency, low‐voltage EPS‐training (d30–d40) may modulate the morphological and functional features of NMJs without inducing broad transcriptional changes. In contrast, on day 60, we detected significant upregulation of 46 genes in EPS‐NMOs subjected to stable pacing and of 52 genes in EPS‐NMOs subjected to dynamic pacing (29 of those genes were common in both groups), while in the latter a significant downregulation of 4 genes was also detected. Functional enrichment analysis on day 60 correlated the upregulated genes with several cellular processes, in both neural and muscle NMO tissues, including neuronal and dendrite function, axon guidance, myelination, muscle contraction, and myogenesis (Figure S11a).

In both groups, this was driven primarily by a marked increase in the expression of *ARHGEF15, COL5A3, COL4A1, and ITGA1* over time, which is more prominent from day 40 to day 60 (Figure S10b). *ARHGEF15*, coding for RhoA guanine nucleotide exchange factor, Ephexin5, has been implicated in physiological spatiotemporal development and maturation of synapses [64]. Although this gene has been studied in the context of synaptic development in the brain, other members of the Ephexin‐protein family (e.g., Ephexin1) have been reported to regulate postsynaptic maturation at NMJs [65]. Genes encoding collagen type IV and V and associated receptors (*COL5A3, COL4A1, ITGA1*) play an essential role in Extracellular Matrix (ECM) structural support and intercellular communication [66, 67], including ECM‐orchestrated NMJ organization and long‐term maintenance, neuronal connectivity, as well as in myogenesis and satellite cell activation [68]. Moreover, we found that the highly significant and positive fold‐change *of ITGA1* expression was also linked to improved muscle contraction in both EPS‐NMO groups, validating our earlier findings on chronic EPS‐boosted NMO functionality. Notably, neuronal and axon functions, along with muscle contraction and myotube formation, were also enriched by significant positive fold‐changes in the expression of other genes (e.g., *ANXA1, MYH9, EGR1, and COL4A2)* in EPS‐NMOs trained under stable parameters (Figure S11a,b), further explaining the prominent effect on NMO spontaneous contractile activity we observed in this group.

Overall, these findings indicate that EPS training can effectively drive neuromuscular maturation, mainly through enrichment of functional pathways, while preserving NMO identity. Additionally, differential effects of stable vs. dynamic pacing parameters on EPS‐NMO transcriptomic profile highlight the robustness and flexibility of this approach in controlling and guiding several cell/tissue properties and functions according to desired outcomes by simply tailoring the electrical pulse parameters.

### 2.5 Dynamic Maturation of NMO Neural Tissue During Chronic Electrical Pulse Stimulation Training

Having established a robust framework for chronic EPS training of NMOs, we then shifted our focus to tissue‐specific effects. First, we assessed neural maturation using immunofluorescence analysis on day 40 (Early‐stage NMOs) and day 60 (Chronic EPS, *stable* and *increasing* NMOs).

To examine the effects of EPS on the proliferation of neuronal progenitors, we co‐labeled samples for SOX1 and Ki67 and quantified their expression at pre‐determined timepoints (Figure S12a). As expected, over time and as NMOs grow, the proliferation of neural progenitors decreased. Chronic EPS training further reduced the Ki67^+^ cell population in the neural compartment on day 60, which was significant only in the Chronic EPS, *increasing* group, while the trajectory of the SOX1^+^ cell population remained similar to non‐paced control NMOs (Figure S12b).

**FIGURE 3:**
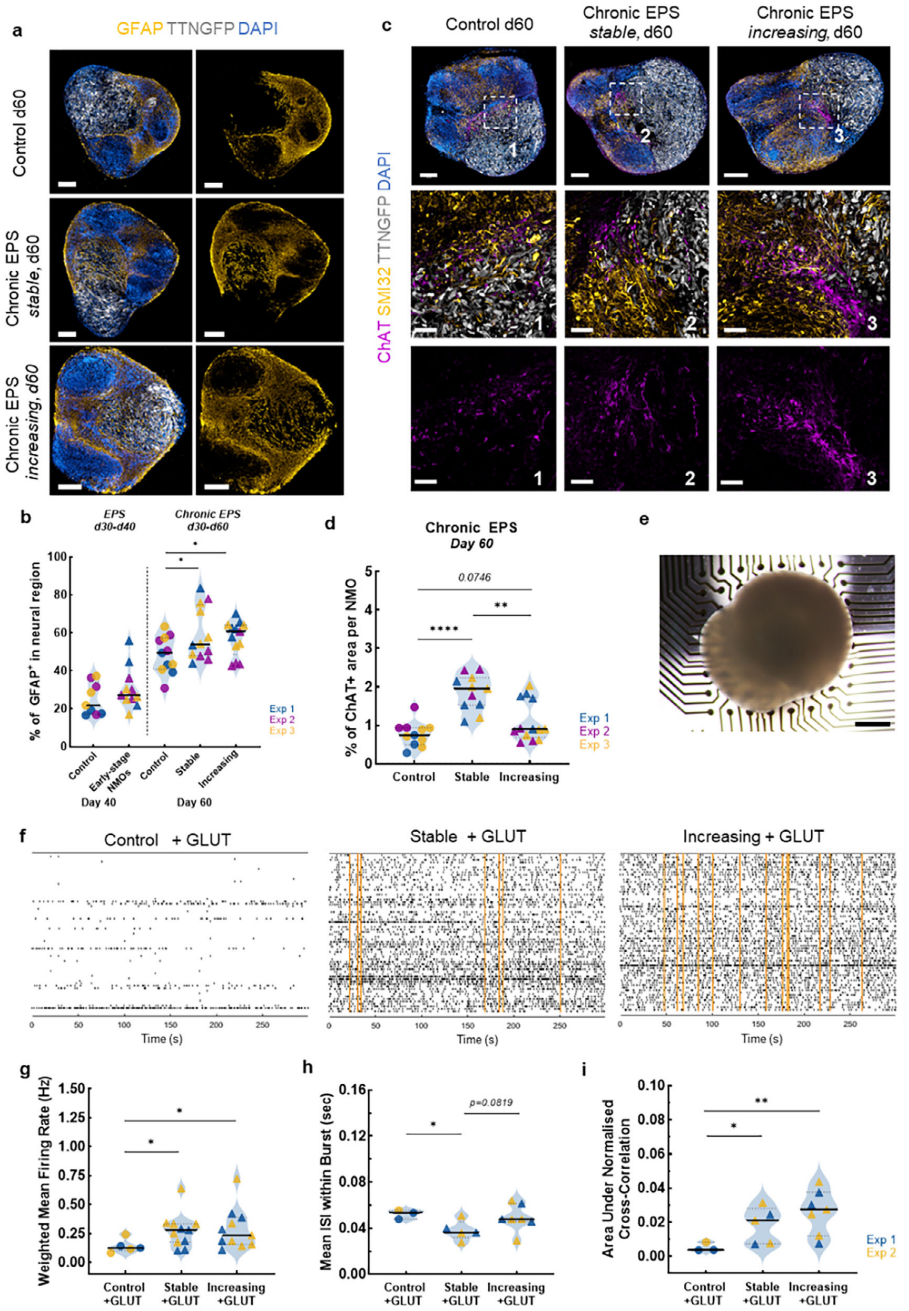
High‐content, high‐resolution imaging and evaluation of the EPS training effects on NMO neural tissue maturation. (a) Representative high‐content, high‐resolution images of whole NMO sections on day 60, illustrating the presence of glial fibrillary acidic protein positive cells (GFAP, in yellow) in WTC^mTTNGFP^ non‐paced control and EPS‐NMOs, trained chronically under stable or increasing pulse parameters. Scale bar 200 µm. (b) Quantification of the expression of GFAP^+^ glia cells in the neural area of WTC^mTTNGFP^ NMOs. The plot illustrates the GFAP^+^ cell expression over time in non‐paced control NMOs and in EPS‐NMOs on day 40 (Early‐stage) and on day 60 (chronic EPS‐NMOs, trained under stable or increasing pulse parameters). Each datapoint represents one NMO. Data from *N* = 3 independent experiments are analyzed by unpaired *t*‐test with Welch correction (control: day 40 *n* = 9, day 60 *n* = 11 NMOs; Early‐stage: day 40 *n* = 11 NMOs; Chronic EPS, stable: day 60 *n* = 12 NMOs, increasing: day 60 *n* = 13 NMOs; ^*^ *p* ≤ 0.05; ^**^ *p* ≤ 0.01; ^***^ *p* ≤ 0.001; ^****^ *p* ≤ 0.0001). (c) Representative high‐content, high‐resolution images of whole WTC^mTTNGFP^ NMO sections (top panel) on day 60, depicting the presence of spinal cord motor neurons expressing the SMI32 neurofilament marker (in yellow) and/or the acetylcholine (ACh)‐synthesizing enzyme choline acetyltransferase (ChAT; in magenta). Scale bar 200 µm. Bottom panels are higher‐magnification images of the respective annotated areas in whole NMO micrographs of the top panels, highlighting the organization and localization of SMI32^+^ with ChAT^+^ neurons, in regions where NMO neural and muscular segments interface. Scale bar 50 µm. (d) Quantification of the expression of ChAT^+^ motor neurons in day 60 WTC^mTTNGFP^ control and EPS‐NMOs. Each datapoint represents one NMO. Data from *N* = 3 independent experiments are analyzed by unpaired *t*‐test with Welch correction (control: *n* = 11 NMOs; stable: *n* = 11 NMOs; increasing: *n* = 13 NMOs; ^*^ *p* ≤ 0.05; ^**^ *p* ≤ 0.01; ^***^ *p* ≤ 0.001; ^****^ *p* ≤ 0.0001). (e) Brightfield image of a day 60 NMO placed on the recording area of a 6‐well MEA plate. (f) Representative Raster plots displaying the spike and neuronal network traces during 5‐min recordings of glutamate‐induced (25 µM) activity of control and EPS‐NMOs on day 60. (g) Plot illustrating the Mean Firing Rate (Hz), normalized to the number of active electrodes (weighted), of the experimental group in response to glutamate (25 µM) stimulation (control: *n* = 5 NMOs; stable: *n* = 11 NMOs; increasing: *n* = 11 NMO). (h) Mean Interspike Interval (ISI) between burst events (in seconds) of each experimental group NMOs in g that exhibited bursting events in response to glutamate. (i) Plot of the area under the normalized cross‐correlation plot, commonly used as a measure of neural network synchrony. (h, i): (control: *n* = 3 NMOs; stable: *n* = 5 NMOs; increasing: *n* = 7 NMOs; (g–i): data from *N* = 3 independent experiments are analyzed by unpaired *t*‐test with Welch corrections; ^*^ *p* ≤ 0.05; ^**^ *p* ≤ 0.01; ^***^ *p* ≤ 0.001; ^****^ *p* ≤ 0.0001).

To evaluate astrocyte maturation, we analyzed Glial Fibrillary Acidic Protein (GFAP) expression [69]. As clearly seen in representative micrographs in Figure 3a and Figure S13a, GFAP^+^ cells increased over time, both in non‐paced control NMOs and in EPS‐NMOs, as expected. Specifically, on day 40, GFAP^+^ astrocyte clusters localized in the central region of the NMOs, close to the neural‐muscle tissue interface, while on day 60, an extensive GFAP^+^ cell network could be observed in the neural NMO compartment. In fact, quantification of GFAP^+^ area confirmed a time‐dependent expansion of astrocytic populations (d40 vs. d60) and showed a significantly greater increase in the neural regions of day 60 EPS‐NMOs, subjected to both stable and progressive pacing, compared to non‐paced controls (Figure 3b and Figure S13b).

Next, by further leveraging high‐content, high‐resolution imaging, we examined the effects of EPS training on motor neuron maturation. We have previously demonstrated that NMOs contain spinal cord motor neurons expressing SMI32 neurofilament marker and Choline Acetyl Transferase (ChAT) enzyme, responsible for the biosynthesis of ACh [48]. EPS‐NMOs exhibited extensive expression of SMI32 by motor neurons that project axons in the skeletal muscle regions, along with enhanced expression and clustering of ChAT^+^ motor neurons at the neural‐muscle interface, consistent with an EPS‐driven enhanced maturation status (Figure 3c). Quantitative analysis revealed a significant increase in ChAT expression, specifically in NMOs subjected to chronic EPS‐training under stable parameters (Figure 3d).

We then evaluated the presence and spatial distribution of Schwann cell populations in EPS‐NMOs. In line with our previous findings, high‐content, high‐resolution immunofluorescence analysis revealed that S100β^+^ cells are highly localized at the neural‐muscle region interface (Figure S13c). This pattern appears more pronounced in both EPS‐trained groups, with increased S100β^+^ projections in the muscle region. Representative images show S100β^+^ cells in close proximity to α‐ΒΤΧ^+^ AChR clusters at NMJ‐like structures, consistent with the spatial distribution expected for terminal Schwann cells. Quantification of α‐ΒΤΧ^+^ and S100β^+^ NMJ‐associated regions showed a trend toward increased numbers in EPS‐NMOs trained under stable pulse parameters, whereas no significant change was observed under increasing‐parameter stimulation (Figure S13d). To further validate these findings, we examined the spatial association of α‐ΒΤΧ^+^ AChR clusters with SOX10, as another, more specific, terminal Schwann cell marker [70]. Quantitative analysis revealed increased SOX10^+^ and α‐ΒΤΧ^+^ NMJ‐associated regions in both EPS‐NMO groups, reaching statistical significance in EPS‐NMOs trained under stable parameters (Figure S13e). Together, these findings support that chronic EPS‐training promoted the association of terminal Schwann cells with NMJ‐like regions, consistent with enhanced neuromuscular maturation.

In the next step, we evaluated the effects of chronic EPS training on neuronal activity in NMOs using multi‐electrode array (MEA) recordings (Figure 3e). Upon acute Glutamate stimulation (25 µM), EPS‐NMOs exhibited more organized and synchronous electrical activity, compared to the relatively sparse electrical activity of control NMOs, as seen in the representative traces/raster plots (Figure 3f). The mean firing rate of EPS‐NMOs was also significantly increased (∼2‐fold), compared to control NMOs (Figure 3g). Although the burst frequency and number of spikes per burst were similar between control and EPS‐NMO groups (Figure S13a), the mean inter‐spike interval (ISI) between bursts was significantly reduced in EPS‐NMOs trained under stable pulse parameters (Figure 3h), suggesting enhanced neuronal excitability and more efficient excitatory synaptic transmission. Consistent with this, we found a marked increase in synchronous firing across different EPS‐NMO regions, reflected by a significant increase in the area under the curve of the normalized cross‐correlation graphs (Figure 3i and Figure S14b). These findings demonstrate that chronic EPS‐training promotes the formation of more active, highly connected neuronal networks in NMOs.

Additionally, we assessed NMO neural tissue functionality by glutamate‐induced calcium imaging. Due to the large size of the organoids, recordings were performed in neurons located at the outer NMO regions, following short‐term adherence to Matrigel‐coated imaging plates, enabling live imaging of NMOs at single‐neuron resolution. Calcium transients revealed synchronous firing events across all groups (Figure S14c and Movies S20–S22), while quantitative analysis of kinetic parameters showed differences in calcium dynamics between control and EPS‐NMOs (Figure S14d). EPS‐NMOs trained under stable parameters exhibited similar calcium buffering kinetics to controls (*Time‐to‐peak (sec)* and decay *Time from Max ΔF/F_0_ to F_0_ (sec)*), but reduced calcium transient amplitudes (*Max ΔF/F_0_*), indicating altered neuronal responsiveness and network activity. Such patterns have been associated with more regulated and refined network behavior in more mature neural circuits [24, 71, 72]. In contrast, EPS‐NMOs trained under increasing parameters exhibited similar peak amplitudes, but significantly prolonged decay times, a feature commonly associated with less mature neuronal states (Figure S14d). Given the spatial constraints of imaging in large organoids, these findings should be interpreted as indicative of changes in neuronal dynamics rather than definitive measures of synaptic maturation.

Collectively, the morphological and functional characterization of NMOs revealed a positive effect of chronic EPS‐training on neural tissue maturation. This is supported by the increased expression of GFAP^+^ and ChAT^+^ cells in EPS‐NMOs, along with enhanced association of terminal Schwann cells with NMJs. These changes coincide with improved neural activity and network dynamics in response to glutamate stimulation. MEA recordings revealed increased neuronal excitability and synchrony in EPS‐trained NMOs, consistent with more active and connected neural networks. Calcium imaging analysis further identified differences in activity dynamics between electrical stimulation conditions. Notably, EPS‐NMOs trained under stable parameters exhibited a distinct calcium response profile compared to controls, whereas EPS‐NMOs trained under increasing parameters showed prolonged decay kinetics. Taken together, these findings support that chronic EPS modulates neural network properties and promotes functional maturation of NMOs, while different pulse stimulation waveforms differentially influence aspects of neural activity.

### 2.6 Dual Role of Chronic Electrical Pulse Stimulation in Skeletal Muscle Maturation

Electrical stimulation of skeletal muscle tissue in in vivo and in vitro models is known to promote myoblast fusion and myogenic differentiation through satellite cell activation [73, 74]. To test whether EPS exerted similar effects in our study, we performed high‐content, high‐resolution quantitative image analysis of muscle progenitor and differentiation markers on day 40 and day 60 EPS‐NMOs.

**FIGURE 4:**
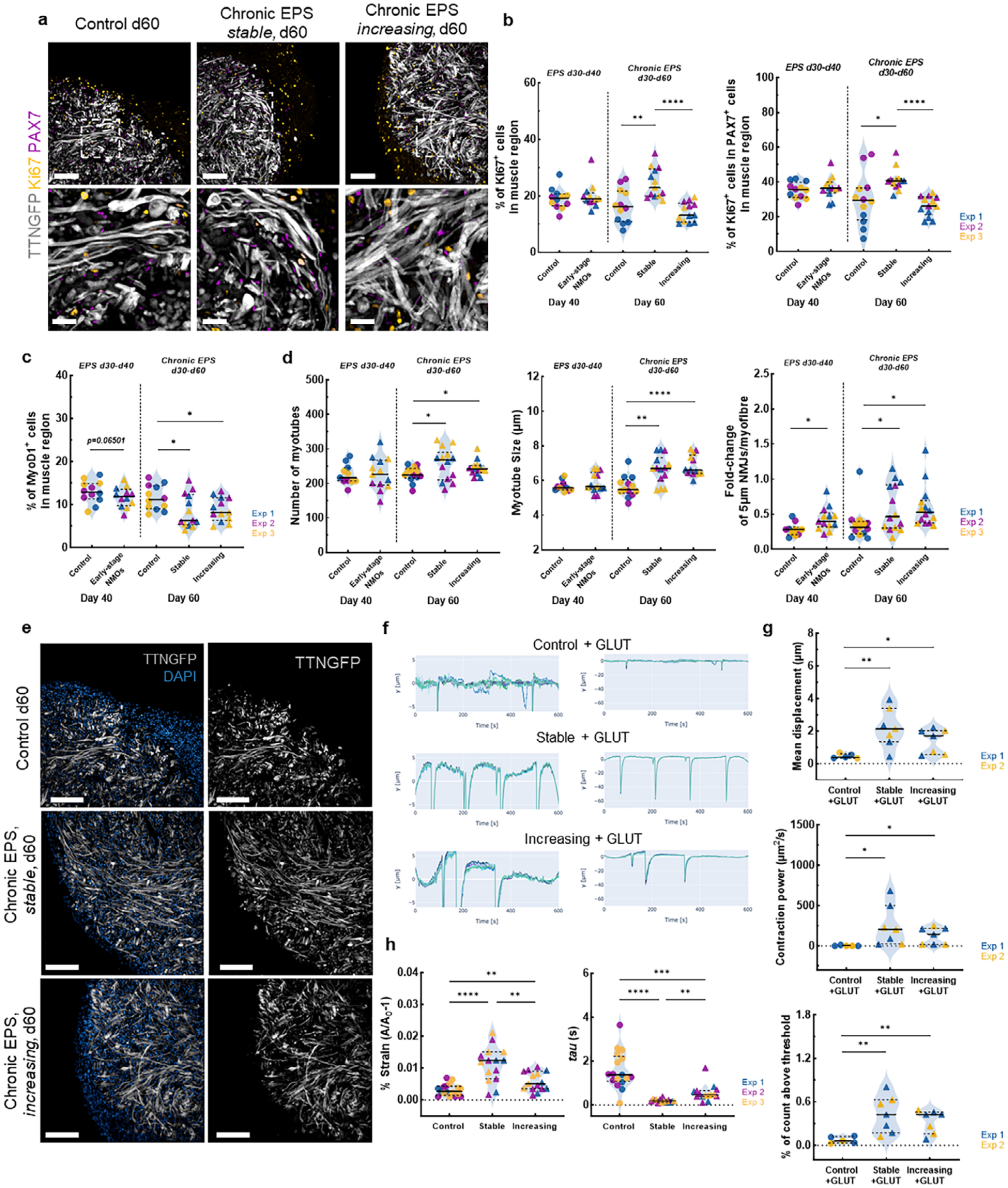
High‐content imaging and quantitative analysis of the EPS training effects on NMO muscle tissue maturation. (a) Representative 20× images (top panel; scale bar 100 µm) and respective magnified renderings (bottom panel; scale bar 25 µm), offering a close‐up look at the NMO muscle fiber organization and localization with satellite‐like PAX7^+^ (in magenta) and/or proliferating Ki67^+^ (in yellow) cells in WTC^mTTNGFP^ non‐paced control NMOs and EPS‐NMOs. (b, c) Quantification of the expression PAX7^+^, Ki67^+^ cells, and myogenic progenitors (MyoD1^+^) in the NMO muscle region over time (d40–d60), between WTC^mTTNGFP^ non‐paced control NMOs and EPS‐trained NMOs. Each datapoint represents one NMO (control: day 40 *n* = 12, day 60 *n* = 12 NMOs; Early‐stage: day 40 *n* = 12 NMOs; Chronic EPS, stable: day 60 *n* = 13 NMOs, increasing: day 60 *n* = 13 NMOs). (d) Quantification of myotube and myofiber features. Plots depict changes over time (d40–d60) in the absolute number of myotubes, in myotube size, and in NMJ > 5 µm number per myofiber in WTC^mTTNGFP^ non‐paced control NMOs and EPS‐trained NMOs. Each datapoint represents the mean number of myotubes per three 63× micrographs and the mean size of myotubes per one 63× micrograph per NMO. The mean ratio of NMJ > 5 µm/myofiber per three 63× micrographs was used for calculating the respective fold‐change (control: day 40 *n* = 11, day 60 *n* = 13 NMOs; Early‐stage: day 40 *n* = 13 NMOs; Chronic EPS, stable: day 60 *n* = 14 NMOs, increasing: day 60 *n* = 14 NMOs). (e) Representative 20× images of d60 WTC^mTTNGFP^ NMO muscle region sections. Inherent expression of Titin protein (TTNGFP; pseudo‐colored gray) demonstrates the organization of the muscle sarcomere with typical striation, as well as muscle fiber fusion and alignment. DAPI nuclear staining also highlights peripheral nucleation and the presence of multi‐nucleated fibers. Scale bar 100 µm. (f) Representative *Time Series* plots and corresponding small‐scale insets of glutamate‐induced (25 µM) contractile response of all NMO experimental groups. (g) Quantitative analysis of contractile outputs of data in e. Each dot represents one NMO. Data from *N* = 2 independent experiments are analyzed by unpaired *t*‐test with Welch correction (control: *n* = 5, Chronic EPS, stable: *n* = 7, increasing: *n* = 7; ^*^ *p* ≤ 0.05; ^**^ *p* ≤ 0.01; ^***^ *p* ≤ 0.001; ^****^ *p* ≤ 0.0001). (h) Mechanobiological properties of WTC^mTTNGFP^ EPS‐NMOs on day 60, compared to non‐paced control NMOs. The plot in the left panel illustrates the maximum change in muscle region area (*A*/*A_0‐1_*) due to tissue deformation during contraction (*% of Strain*), as a contraction strength index. The plot in the right panel depicts relaxation dynamics, expressed as relaxation time constant (*tau*), as a tissue stiffness index (control: *n* = 17 NMOs; Chronic EPS, stable: *n* = 15 NMOs, increasing: *n* = 15 NMOs). In all plots, data from *N* = 3 independent experiments are analyzed by unpaired *t*‐test with Welch correction (^*^ *p* ≤ 0.05; ^**^ *p* ≤ 0.01; ^***^ *p* ≤ 0.001; ^****^ *p* ≤ 0.0001).

Co‐labeling of NMOs for Paired Box 7 (PAX7) transcription factor and Ki67 proliferation marker revealed a highly proliferative PAX7^+^/Ki67^+^ cell population on day 60 EPS‐NMOs trained under stable conditions (Figure 4a,b and Figure S15a,b). Moreover, consistent with continuous NMO maturation, the expression of Myoblast Determination protein 1 (MYOD1), which regulates myoblast differentiation and maturation [75], decreased from day 40 to day 60. In line with prior literature [76, 77], this trend was more pronounced in EPS‐NMOs, which on day 40 already exhibited lower MyoD1 levels that continued to decrease and, by day 60, were significantly reduced compared to non‐paced control NMOs (Figure 4c and Figure S16). These trends suggest that EPS‐training promotes a shift in myogenic population dynamics, characterized by reduced MYOD1^+^ cells alongside maintenance or expansion of proliferative PAX7^+^ cells, consistent with activity‐dependent muscle adaptation.

We further examined the effects of EPS on NMO muscle formation and maturation, based on immunofluorescence analysis of Fast Myosin Heavy Chain (Fast MyHC) expression. Quantification of absolute myotube number and size (diameter) revealed a significant increase in both features on day 60 EPS‐NMOs, under either stable or increasing pulse parameters, but not after early, acute/short‐term stimulation (d30–d40; Figure 4d and Figure S17a). However, when we assessed the number of mature NMJ‐like structures (>5 µm), normalized for the number of muscle fibers – a key marker during neuromuscular development – we found a significant progressive increase between day 40 and day 60, compared to non‐paced control NMOs (Figure 4d). The trend was similar in newly‐formed NMJ‐like structures (>2 µm) and consistent across both EPS‐NMO groups (Figure S17b), validating our early observations about enhanced NMJ‐like structure morphology and function upon electrical stimulation training.

Despite the increase in the number and size of myotubes, overall muscle tissue proportion remained largely unchanged. On day 40, muscle area accounted for 46% of total NMO size and remained stable in control NMOs until day 60. EPS‐NMOs trained under stable parameters displayed a modest ∼5% increase (∼51% of NMO size is “occupied” by muscle tissue), which was significant only compared to the increasing‐parameter condition, but remained comparable to controls (Figure S17c). These findings indicate that EPS primarily enhances tissue maturation without substantially altering overall tissue composition.

We further examined the maturation status of both NMJ‐like structures and muscle fibers by gene expression analysis of key NMJ structural features and myosin isoforms. As transcriptomic shifts were primarily observed under chronic EPS‐training, we focused our analysis on day 60 NMOs. Specifically, analysis of *CHRNE* and *CHRNG* genes, encoding AChR epsilon (ε) and gamma (γ) subunits, respectively, revealed a chronic EPS‐induced γ‐to‐ε AChR subunit switch (Figure S18a). Albeit significant only for EPS‐NMOs trained under increasing parameters, this subunit switch is consistent with NMJ maturation [44, 47]. Furthermore, quantification of gene expression levels of *MYH3* (fetal), *MYH4* (more mature), and *MYH2* (fast, more mature) showed increased ratios of mature to fetal isoforms (*MYH2/MYH3* and *MYH4/MYH3*) in chronic EPS‐NMOs, as expected. These changes were again more pronounced under increasing stimulation parameters, where they reached statistical significance (Figure S18b) and are consistent with activity‐dependent muscle maturation [41, 45, 78, 79].

Additionally, we evaluated the NMO muscle tissue morphology qualitatively, leveraging our NMO TTNGFP^+^ inherent signal. Figure 4d illustrates representative 20× field‐of‐view (FOV) micrographs of NMOs on day 60, derived from high‐content, high‐resolution imaging. What is strikingly evident in these images is the dramatic increase in the length of TTNGFP^+^ muscle fibers in the chronic EPS conditions compared to non‐paced controls, indicating enhanced myotube formation.

We next studied how changes in NMO muscle and NMJ properties relate to tissue functionality. To this end, we monitored the contractile activity of NMOs in response to acute Glutamate stimulation (25 µM). Qualitative and quantitative analysis of contraction properties (Figure 4e,f; see also Movies S23–S25) revealed a more synchronous and stronger contractile response of NMO muscle tissue following glutamate stimulation. Both EPS‐trained NMO groups exhibited increased contraction frequency (*% of count above threshold*), more pronounced displacement (*Mean displacement* (µm)), and higher contraction magnitude (*Contraction power* (µm^2^ s^−1^)), compared to control NMOs, in line with our phenotypic and transcriptomic analyses. Specifically, functional gene enrichment analysis correlated improved muscle contraction with upregulation of ECM‐protein encoding genes in EPS‐NMOs. Muscle tissue is known to respond to physical stimuli by remodeling its ECM, as a means to handle increased strain, including enrichment of collagen IV within the basal lamina, which has been linked to more efficient ECC, while protecting muscle fibres from potential damage [79, 80]. We therefore examined whether the increased expression of collagen IV (COL4A1) detected by RNA‐seq was reflected at the protein level. Quantitative immunofluorescence image analysis of day 60 NMOs revealed significantly higher COL4A1^+^ areas in EPS‐NMOs trained under stable parameters, compared to control, with strong localisation of the signal in the muscle NMO region, particularly within the inter‐fiber space (Figure S19). Notably, increased expression of collagen IV signal was also detected in the neural region of these NMOs. Similar trends were observed for collagen V (COL5A3; Figure S20), although these changes did not reach statistical significance. These findings are consistent with our transcriptomic data and support ECM remodeling associated with EPS‐induced functional neuromuscular maturation.

Finally, we examined whether EPS‐induced upregulation of ECM proteins, along with improved contractile properties, had any effects on the mechanobiological maturation of NMOs. To this end, we quantitatively analyzed the strain index and relaxation time of stimulation‐induced contractions during time‐lapse recordings (Figure S21). Both non‐paced control samples and EPS‐NMOs exhibited reproducible responses to electrical pulses (1 Hz, 10 V, 10 ms), as evidenced by muscle region contractions, followed by exponential relaxation toward baseline (Movies S26–S28). However, after one month of training, both groups of EPS‐NMOs exhibited significantly higher peak strain amplitude (% strain: *max peak A/A_0‐1_*) and significantly lower *tau* values (faster relaxation after stimulation‐induced contraction), compared to non‐paced control NMOs (Figure 4g). The increase in the strain index suggests improved force transmission and ECC that support stronger NMO muscle contractions, which can be attributed to more mature muscle fibers with enhanced morphology and innervation, as shown earlier. The marked decrease in *tau* values in EPS‐NMOs, which is more significant in EPS‐NMOs trained under stable parameters, reflects a more rapid relaxation following contraction, which is consistent with the development of stiffer tissues with enhanced viscoelastic properties. In contrast, non‐paced control NMOs retained higher *tau* values, as expected from more compliant and less mechanically mature tissues. We note that tissue stiffness reflects a bulk mechanical property of the organoid, whereas strain index and relaxation dynamics are derived from time‐resolved deformation during electrically evoked contractions and therefore report on dynamic mechanical output. These readouts are complementary, but not equivalent. Together, these findings suggest that EPS‐NMOs undergo mechanobiological maturation, acquiring properties of stiffer tissues, capable of stronger and faster contractions.

Collectively, the morphological and mechanobiological analysis of NMO skeletal muscle tissue further substantiates our findings on EPS‐guided maturation, emphasizing the importance of the prolonged duration of EPS training. Importantly, in our study, the nature of electrical pulse parameters emerged as a key factor, indicating that stable vs dynamic pulse parameters can activate distinct signaling pathways (e.g., PAX7^+^ cell proliferative capacity/status and *tau* values), thereby highlighting the versatility of this method in modulating and controlling tissue properties according to desired outcomes, on demand and in a non‐invasive and non‐destructive way.

## 3 Discussion and Conclusion

Organoids have emerged as highly human‐relevant models and are increasingly used in biomedical research to study development, disease, and therapeutic responses, with growing potential for applications in drug discovery and translational studies. However, recapitulating adult‐like tissue maturation stages in organoids remains a major challenge. Significant progress has been made recently to address this challenge in synergy with bioengineering approaches. Biomechanical loading and/or electrical stimulation have emerged as key drivers for functional maturation of bioengineered tissues [30, 31, 81, 82].

While electrical stimulation can affect neuronal and muscle tissue independently, here, we used complex human NMOs, which enable the study of activity‐dependent responses within an integrated neuromuscular system, and established a framework for chronic, low‐frequency EPS as a training paradigm to enhance maturation of complex organoids. In this context, while EPS can directly activate muscle independently of synaptic transmission, our combined neuronal stimulation, MEA recordings, and NMJ structural analysis indicate that EPS enhances both neuromuscular coupling and muscle intrinsic ECC properties. This framework allowed us to treat EPS not as a single perturbation, but as a systemic training paradigm, in which electrical pacing during distinct developmental windows was used to examine how activity influences the trajectory of NMJ formation and function. Time‐matching the delivery of EPS training to the onset of synaptogenic programs in NMOs proved to be an important determinant. We found that applying EPS from early NMO developmental stages has a more prominent effect on NMJ morphological features, supporting strong and enduring muscle contractions, even upon acute/short‐term EPS training. In contrast, no significant effects were observed on the NMJ‐associated features of “older” NMOs (i.e., Late‐stage d50‐60) exposed to the same pacing cues, despite improved spontaneous contractile activity. These findings suggest a stage‐dependent effect: in Early‐stage NMOs, EPS training may promote synaptogenic processes, potentially by enhancing the AChR clustering at the onset of NMJ formation [83, 84], while at later stages, when NMOs have reached more advanced NMJ maturation states, EPS training is less likely to influence synaptic formation. Rather, it may act partially independently of NMJ activity, as NMOs respond to EPS even upon NMJ blockage, primarily through enhancement of ECC in skeletal muscle, while also acting in conjunction with the already established NMJ networks, potentially by increasing engagement of AChR clusters and thereby strengthening the existing motor neuron‐muscle interactions. Thus, our data indicate that functional improvements in EPS‐NMOs likely arise from a combination of NMJ structural maturation and direct muscle‐intrinsic adaptations, rather than exclusively from enhanced synaptic transmission.

The next factor we identified as a key determinant for effective NMO maturation is the duration of EPS training. Chronic low‐frequency EPS for ∼1 month enhanced NMO maturation, modulating tissue‐specific cellular processes and improving functional neuromuscular output. This enhanced functional capacity correlated with upregulation of transcriptional programs linked to neuromuscular maturation in EPS‐NMOs, without altering overall organoid identity. Notably, these effects were observed under both stable and dynamic pacing conditions, albeit via activation of different transcriptomic pathways, indicating that, by tailoring EPS parameters, it is possible to guide tissues toward desired outcomes.

Quantitative image analysis further resolved tissue‐specific EPS effects. In the neural compartment, electrical stimulation led to a significant increase in glial cell populations [85] and enhanced the localization of motor neurons at the neural‐muscle region interface, consistent with synaptic support and maturation. Functional analyses further point toward EPS‐induced changes in neural activity, as evidenced by enhanced neuronal responsiveness and network dynamics. In muscle tissue, the effects of EPS were multi‐fold. EPS‐NMOs exhibited significantly lower levels of MYOD1, consistent with a shift toward more mature muscle fibers [76, 77], compared to control NMOs. In line with these findings, myofibers in EPS‐NMOs were significantly larger, more elongated, and better aligned, also containing higher numbers of NMJ‐like structures with larger AChR clusters (>5 µm). EPS‐induced myofiber and NMJ‐like structure maturation was accompanied by a γ‐to‐ε AChR subunit switch and modest changes in fetal‐to‐mature myosin isoform expression. Moreover, in line with literature, chronic EPS under stable pulse parameters increased myogenic activity [73, 74], leading to NMOs with significantly more myotubes and higher levels of proliferative PAX7^+^ cells, reflecting our transcriptomic enrichment for ECM and myogenic pathways. Although we cannot fully exclude a degree of microinjury upon electrical pacing (similar to physiological injury of skeletal muscle during exercise in vivo), the molecular profile observed in EPS‐NMOs is more consistent with an integrated adaptive program, involving activity‐dependent remodeling of the stem cell niche and maintenance of regenerative capacity [86, 87]. These findings suggest that controlled EPS paradigms may also provide a useful framework for studying mechanisms of skeletal muscle adaptation and regeneration.

Finally, we also found that EPS‐NMO muscle tissue undergoes mechanobiological maturation, acquiring properties of increased stiffness, capable of stronger contractions and faster relaxation, in line with studies of EPS‐training of skeletal muscle models [38, 42, 57]. These effects were more pronounced in EPS‐NMOs trained under stable parameters and may be linked to enhanced ECM deposition and remodeling, as suggested by increased expression of genes encoding collagen and laminin receptors detected in this group, and significantly increased expression of collagen IV. Importantly, this analysis complements and further validates our earlier findings on the capacity of EPS‐NMOs for stronger spontaneous contractions (i.e., *Mean displacement* (µm)*, Contraction power* (µm^2^ s^−1^)), showcasing the power of our live imaging‐based, non‐destructive, and automated quantification pipeline for evaluating NMO contractile function.

This work represents a proof‐of‐principle study conducted in two different genetic backgrounds of human iPSC lines. Although key phenotypes were reproduced in both WTC^mTTNGFP^ and KOLF2.1J backgrounds, broader genetic diversity and replication across independent lines and donors will be required to establish generalization of the approach. Additionally, the current EPS training protocol remains labor‐intensive and is not yet scalable to very large numbers of NMOs, limiting its application in high‐throughput approaches. Moreover, we relied on bulk RNA‐seq rather than single‐cell RNA‐seq, and therefore, cell‐type‐specific transcriptional programs remain unresolved. Additionally, while EPS induced transcriptional changes consistent with maturation, these changes were relatively modest, suggesting that enhanced neuromuscular function may primarily arise from structural adaptations of the neuromuscular system, rather than large‐scale transcriptional rewiring. Future studies combining EPS with targeted perturbations and single‐cell approaches will help further dissect the relative contributions of transcriptional and structural mechanisms. In addition, development of NMP‐derived reductionist systems (e.g., neural‐only or muscle‐only organoids) may help decipher tissue‐specific responses to EPS, which was not possible here as NMOs generate both neural and mesodermal derivatives within the same self‐organizing system.

Overall, here, we show that EPS can promote the maturation of complex self‐organizing multi‐tissue organoids. Our findings highlight the potential of electrical stimulation in modulating tissue properties in a non‐invasive and non‐destructive manner. We anticipate that the versatility of this approach will enable broader applications, facilitating morphological and functional maturation of diverse organoid systems as human‐relevant models of physiology and disease. To this end, integrating advanced bioengineered approaches will enable on‐demand, multi‐modal monitoring and stimulation of organoids with high spatiotemporal resolution, thereby advancing mechanistic understanding of human neuromuscular system development. In parallel, the development of automated, high‐throughput, and user‐friendly EPS compatible platforms will be important to fully realize its potential in scalable applications. Finally, the integration of electrical stimulation in neuromuscular disease modeling and drug screening applications represents a promising direction for the field.

## 4 Experimental Section

### 4.1 Generation of Neuromuscular Organoids

#### 4.1.1 Human Induced Pluripotent Stem Cell Lines

WTC‐11 (GM25256) hiPSCs, fluorescently tagged with mEGFP to target titin (TTN) protein (WTC^mTTNGFP^, AICS‐0048, obtained from Allen Cell Collection [50]) and KOLF2.1J hiPSCs, (HPSI0114i‐kolf_2, JIPSC001000, obtained from the Jackson Laboratory [49]; Table S1) were used for the generation of neuromesodermal progenitors and neuromuscular organoids, following the protocol described by Martins et al. (2020) [48]. Both cell lines have been QC tested and the respective certificates of analysis were obtained from the providers [49, 50]. hiPSCs were passaged at least twice before being used for experiments in this study (WTC^mTTNGFP^ passage 47 + 4 and KOLF2.1J P3^2^ +8). hiPSCs and NMOs were tested monthly for Mycoplasma. Here, we directly used cryopreserved NMPs of the same bank/batch and performed at least three independent differentiations of NMPs into neuromuscular organoids for WTC^mTTNGFP^ and two differentiations for KOLF2.1J. A full list of reagents and consumables is available in Supplementary Information.

#### 4.1.2 Generation and Cryopreservation of NMPs

iPSCs were cultured on Geltrex LDEV‐Free hESC‐Qualified Reduced Growth Factor Basement Membrane Matrix (Gibco) and maintained in mTESR1 medium (Stem Cell Technologies), at 37°C, 5%CO2. Upon reaching 70% confluency, iPSCs were dissociated into single cells using Accutase (Sigma–Aldrich). Cell counting was performed using Trypan blue (Gibco, Thermo Fisher Scientific) exclusion assay with Countess 3 automated cell counter (Thermo Fisher Scientific). Cells were then plated (day ‐3) on Geltrex‐coated dishes (50 000 cells/cm^2^) and maintained in neurobasal medium (N2B27) supplemented with Rock inhibitor (10 µM; Tocris Bioscience), CHIR99021 (3 µM; Tocris Bioscience), and bFGF (40 ng/mL; produced in‐house). N2B27 medium is a 1:1 medium of Advanced Dulbecco's Modified Eagle Medium F12 supplemented with 1× N2 (GIBCO) and Neurobasal medium (GIBCO) supplemented with 1× B27 (GIBCO). The final medium was further supplemented with 1× GlutaMAX (GIBCO), BSA fraction V (75 µg/mL; Sigma), 2‐mercaptoethanol (0.1 mM; GIBCO). The following day, Rock Inhibitor was removed and fresh N2B27 medium, supplemented with CHIR99021 (3 µM; Tocris Bioscience) and bFGF (40 ng/mL; produced in‐house), was added to the culture. Cells were then maintained for two more days, with daily media changes (N2B27 supplemented with CHIR99021 (3 µM; Tocris Bioscience) and bFGF (40 ng/mL; produced in‐house). At the end of differentiation, NMPs were washed once with 1X PBS and incubated with Accutase (Sigma–Aldrich) or TrypLE (Gibco, Thermo Fisher Scientific) for 5 min at 37°C to obtain a single‐cell suspension. To stop the enzymatic reaction, DMEM/F‐12 (Gibco, Thermo Fisher Scientific) was added, and the cell suspension was transferred to a Falcon tube (15 mL). NMPs were centrifuged at 2000 rpm for 4 min at room temperature, the supernatant was removed, and the pellet was resuspended in N2B27 (1 mL). Cell counting was performed as described above. Upon determining the required volume of cryopreservation media, NMPs were resuspended in Bambanker cryopreservation medium (Nippon Genetics) and aliquoted in cryovials. After overnight freezing at −80°C, cryovials were transferred to liquid nitrogen for long‐term storage.

#### 4.1.3 Generation of NMOs

On day 0, cryovials containing cryopreserved NMPs were briefly incubated in a 37°C water bath until just thawed. NMPs were immediately transferred to pre‐warmed DMEM/F‐12, followed by centrifugation at 2000 rpm for 4 min. The supernatant was removed, the cells were resuspended in N2B27 medium, and the cell concentration was determined upon counting, as described above. To generate NMOs, 9000 NMPs/well were plated in ultra‐low binding 96‐well plates (Thermo Fisher Scientific) in neurobasal medium (N2B27;100 µL/well; STEMCELL), supplemented with Rho‐associated protein kinase ROCK inhibitor (50 µM; BioMol GmbH), bFGF (10 ng/mL; produced in‐house), IGF1 (2 ng/mL; Peprotech), and HGF (2 ng/mL; Peprotech). On day 2, fresh N2B27 medium (100 µL), supplemented with IGF and HGF, was added to each well, so that the final concentration of each small molecule was 2 ng/mL for each compound. From day 4 onward, 100 µL of medium was removed and 100 µL of fresh N2B27 medium without additional growth factors was added, and the medium was replenished every other day. On day 10, NMOs were transferred to 60 mm dishes (Sigma–Aldrich; one dish per 96‐well plate) with N2B27 (6 mL) and maintained on an orbital shaker (BINDER) at 75 rpm. Medium was changed every other day by removing 3 mL from each 60 mm dish and adding 3 mL of fresh N2B27 medium. On day 20, each NMO dish was split into three 60 mm dishes, each containing 6 mL N2B27 medium, and media were replenished as above [48].

### 4.2 Electrical Pulse Training of NMOs

NMOs were included in EPS experiments upon quality checks at different developmental stages, including elongation and segregation of the NMO into mesodermal and neuronal compartments between developmental days 5–10. In addition, only NMOs that exhibited clear compartmentalization at the predefined timepoints (day 30, 40, 60, 75) were selected for analysis.

For EPS pacing assays, each NMO experimental group was transferred to dedicated electrical stimulation chambers. Each chamber comprised a well of a six‐well plate, prefilled with fresh N2B27 media (4.5 mL), which was topped up (6 mL) by adding spent media from each NMO group dish (1.5 mL), and a clean C‐Dish graphite electrode circuit board (IonOptix Pace C‐EM; IonOptix/CytoCypher B.V) placed between the well‐plate and its lid. Plates were then transferred to the incubator and were connected to the IonOptix pulse generator dedicated channels, programmed to deliver pulses according to the parameters of each experimental group (Figures 1c and 2a), for 30 min. NMOs were then transferred back to their dedicated 60 mm dishes, replenished with fresh N2B27 media.

The used C‐Dish graphite electrode boards were then thoroughly cleaned, following the manufacturer's protocol, and sterilized under UV for 30 mins before being used again.

### 4.3 Bulk RNA Sequencing

#### 4.3.1 Sample Collection and RNA Extraction

On days 30, 40, and 60, NMOs were collected for RNA sequencing by pooling and snap‐freezing 3–5 NMOs per EPS group and controls. Total RNA per sample was extracted using the Direct‐zol RNA Miniprep Plus Kit (ZYMO RESEARCH), following the manufacturer's guidelines. Samples were stored at −80°C until ready for sequencing.

#### 4.3.2 Library Preparation and Sequencing

Total RNA samples were quantified using a Qubit Fluorometer, and RNA integrity was checked on a TapeStation (Agilent). Double‐indexed stranded mRNA‐Seq libraries were prepared using the NEBNext Ultra II Directional RNA Library Prep with Beads Kit (NEB, # E7765L), starting from 400 ng of input material according to the manufacturer's instructions. Libraries were equimolarly pooled based on Qubit concentration measurements and TapeStation size distributions. The loading concentration of the pool was determined using a qPCR assay (Roche, #7960573001). Libraries were then sequenced on the Illumina NovaSeq X Plus platform using PE100 sequencing mode using a 10B flow cell with the 200‐cycle reagent kit, with a target of 30 million reads per library. Libraries were sequenced on an Illumina NovaSeq X Plus using a 10B flow cell with the 200‐cycle reagent kit. The run was configured for paired‐end, dual‐indexed sequencing with the following cycle scheme: 101–10–10–117 (Read 1—Index 1—Index 2—Read 2). Instrument and reagent specifications for the NovaSeq X Series (including 10B/200‐cycle kits) and the dual‐index workflow are described by Illumina.

#### 4.3.3 Read Processing and Quality Control

Raw FASTQ files were assessed with FastQC (v0.12.1), and summary reports were inspected and collated with MultiQC (v1.25.1). Read‐level quality metrics (per‐base quality profiles, adapter content, duplication) indicated high‐quality libraries with negligible adapter contamination; therefore, no read trimming was applied prior to alignment. FastQC documentation and modules were used as references for interpretation.

#### 4.3.4 Alignment and Quantification

Reads were aligned to the human reference genome GRCh38 (GENCODE v49/Ensembl 115 annotations) using HISAT2 (v2.2.1) with strand‐specific settings (–rna‐strandness FR) and otherwise default parameters. Aligned reads were sorted and indexed with samtools (v1.22.1). Gene‐level counts were derived with featureCounts (Subread v1.5.3‐0) in paired‐end and stranded mode (‐p ‐S 2) against the Ensembl release‐115 Homo sapiens Gene Transfer Format (GTF), producing a matrix of raw counts per gene per sample.

#### 4.3.5 Differential Expression Analysis

Differential gene expression between EPS‐stimulated and unstimulated conditions at days 40 and 60 was performed in R (v4.3.3) using DESeq2 (v1.46.0). Count data were imported without external normalization; DESeq2's median‐of‐ratios method was used for size‐factor normalization, followed by dispersion estimation and fitting of negative binomial generalized linear models. Unless otherwise specified, Wald tests were used for contrasts of interest, and *p*‐values were adjusted for multiple testing using the Benjamini–Hochberg procedure. Genes with FDR < 0.05 and |log2FC| ≥ 1 were considered significantly differentially expressed.

#### 4.3.6 Gene Set Enrichment Analysis

Two complementary enrichment strategies were applied. (i) Over‐representation analysis was conducted with enrichR (v3.4) using MSigDB Hallmark and GO Biological Process databases on significant up‐ and down‐regulated genes (defined by the above‐mentioned thresholds). (ii) Rank‐based enrichment was performed with fgsea (v1.32.4) on preranked genes ordered by DESeq2 Wald statistic using 50 000 permutations. Multiple‐testing correction was applied to all enrichment results (Benjamini–Hochberg), and pathways with FDR < 0.05 were considered significant.

### 4.4 Analysis of NMO Size

Before subjecting NMOs to EPS (day 30, day 50) and at pre‐determined sample collection timepoints, 0.7× brightfield images of NMOs were acquired using a stereoscope (Olympus SZX16). For all analyses, only *.tiff images were used. Images were then processed using an automated pipeline to extract morphological features of the organoids. First, Cellpose, a widely used framework for automated single‐cell segmentation [88], was used to extract a segmentation mask of the organoids. Although Cellpose was originally developed to segment cellular structures, its use has been extended beyond single‐cell segmentation to other round‐like entities [89, 90]. Among the available models, the cyto 3 model was chosen here due to the more robust and accurate segmentation capabilities it offers, compared to earlier models, including image restoration support, denoising, and blurring for enhanced border detection. In Cellpose, all images are by default resized so that all objects have approximately similar diameter (∼30 pixels). Given that our organoids are significantly larger, to enhance the accuracy of the model and guide it to efficiently detect objects at our desired scale, we defined an extra step for adjusting this default diameter, named *average diameter*. This value is determined by the user upon a quick evaluation of the NMOs with the largest and smallest diameters, based on each image scale bar. Subsequently, the image processing library for Python scikit‐image [91] was used to extract the area and the centroid coordinates from the segmented mask. The output of this analysis pipeline is a segmented image and an *.xlsx file (one per image), containing the individual label ID for each organoid, the organoid area in pixels, and the respective centroid coordinates. The user can manually evaluate the segmented image and make adjustments in the average diameter, if necessary, to improve segmentation accuracy or to exclude NMOs unsuitable for analysis. In this study, NMOs that did not exhibit distinct compartmentalization in the neural and muscle region were not included in the downstream analysis. To quantify the NMO size, the area in pixels of each NMO is converted into physical units by the user, based on the scale bar of each image. This manual conversion, properly implemented in the Excel file, allows use of the pipeline in other experimental setups or image formats, which may have different conversion factors, rendering the tool highly flexible and broadly applicable.

### 4.5 Immunofluorescence Analysis of NMOs

#### 4.5.1 Sample Collection, Embedding, and Sectioning

At predefined timepoints for analysis, NMOs were fixed with 4% para‐formaldehyde (PFA; VWR) for 1 hr on ice, followed by thorough washes with 1X PBS before overnight treatment in 30% sucrose. NMOs were then embedded using a warm 15% gelatin‐10% sucrose (Sigma–Aldrich) solution, followed by solidification at 4°C and snap‐freezing in isopentane before storing at −80°C. Organoids underwent cryosectioning in 16 µm thick slices (MicroM HM 560 Cryostat, Thermo Fisher), collected on Superfrost Plus microscope glass slides (epredia).

#### 4.5.2 Immunofluorescence Staining and Imaging

Before staining, NMO sections were incubated in 1X PBS at 42°C for 3 × 30 min to remove gelatin. Sections were then fixed with 4% PFA for 5 min on ice, followed by permeabilization and blocking steps with 4% Bovine Serum Albumin (BSA; VWR) and 0.3% Triton X‐100 (Sigma–Aldrich) for 90 min at room temperature, followed by overnight incubation with primary antibodies at 4°C (Table S2). After thorough washing with 0.3% Triton X‐100 (PBST, 3 × 30 min), samples were incubated with species‐specific secondary antibodies, conjugated with Alexa Fluor 488, 568, 647 (Table S2), for 2 h at room temperature, washed with PBST (3 × 30 min), counter‐stained with DAPI (Thermo Fisher Scientific), and mounted using Immu‐Mount medium (epredia). Samples were then imaged using either a confocal microscope (Leica SP8) or a spinning disc confocal microscope (Opera Phenix Plus, Perkin Elmer).

#### 4.5.3 Image Analysis

Analysis of NMJ‐like structures and myotubes and myofibers was based on 63× confocal micrographs (ROIs; LEICA SP8) of NMO sections co‐labeled for α‐ΒΤΧ, Fast MyHC, and TUBB3. Per NMO section, at least three ROIs were selected within the muscle region, avoiding the outer edges, and distributed across the muscle area to ensure representative sampling. The number of ROIs per section was kept consistent across all sampling timepoints.

The absolute number of myotubes was quantified by manually counting the number of Fast MyHC^+^ myofibers in three 63× micrographs/per NMO, using the multi‐point tool in FiJi ImageJ. Similarly, the size of Fast MyHC^+^ myofibre cross‐sections was quantified in one 63× micrograph/per NMO as the minimum Feret's diameter, using straight line ROI measurements in FiJi ImageJ. NMJ‐like structure number, size, and innervation were analyzed via a custom semi‐automated image analysis pipeline of three 63× micrograph/per NMO, developed with ilastik (version 1.4.0.post1) and Python (version 3.9.21). Ilastik models were trained for α‐ΒΤΧ and TUBB3 images. Both α‐ΒΤΧ and TUBB3 images were segmented with ilastik, and the respective masks were exported as NumPy arrays (*.npy). These were then processed and analyzed in Python, where NMJ‐associated structures were defined as α‐ΒΤΧ^+^ AChR clusters above a defined‐size threshold (>2 µm), and innervated NMJs were defined as those showing spatial overlap with neuronal markers and referred to as NMJ‐like structures. α‐ΒΤΧ^+^ regions, corresponding to postsynaptic specializations, were used to calculate AChR cluster size and absolute number by resorting to the skimage library. The absolute number of AChR clusters was calculated per 63× micrograph. Cluster size was calculated as the length of the major axis of the ellipse that has the same normalized second central moments as the α‐ΒΤΧ^+^ region. TUBB3 masks were used to assess NMJ innervation by expanding them by 2 pixels and overlaying them with the corresponding α‐ΒΤΧ mask. An NMJ was considered innervated if the α‐ΒΤΧ^+^ region overlapped with the expanded TUBB3 mask. Only AChR clusters >2 µm were included in the analysis. The results of this analysis were automatically exported as output data *.xlsx files, along with confirmation images for validation and data analysis. The absolute number of NMJ‐like structures per 63× micrograph was also normalized to the count of myotubes in the respective image to obtain the number of NMJ‐like structure/myofiber. Fold changes were calculated by dividing the NMJ‐like structure number/myofiber calculated for each image at each time point (days 40 and 60) by the mean NMJ‐like structure number/myofiber of control images at the respective time point.

Spinning disc confocal images were used for the analysis of levels of apoptosis, proliferation, neural, and muscular tissue‐specific progenitor and maturation marker expression in whole NMO sections, based on semi‐automated custom image analysis pipelines developed in ilastik and Python. In all cases, the muscle region was detected by Titin fibers, inherently expressing GFP (TTNGFP), unless otherwise stated. Prior to processing, masks of neural, muscle, and/or whole NMO were generated with FiJi, upon deconvoluting regions of interest (ROIs) based on tissue‐specific biomarkers using the free‐hand tool. In some images, GFP is pseudo‐colored white. Ilastik models were trained for several biomarkers of interest.

Apoptotic levels, glial cell population, motor neuron population, and expression of collagen IV and V were quantified in whole NMO sections labeled for cCas‐3, TUNEL, GFAP, ChAT, COL4A1, and COL5A3, respectively, and counterstained with DAPI. In ilastik, cCas‐3 and GFAP signals were segmented, and the respective masks were exported as NumPy arrays (*.npy). Upon processing in Python, apoptosis in neural and muscle regions was calculated as the percentage of cCas‐3 or TUNEL area coverage in each region. The expression of collagen IV and V was quantified with the same approach, based on COL5A3^+^ and COL4A1^+^ areas in each NMO region. Similarly, the glial cell population was calculated as the percentage of GFAP^+^ area in neural and whole NMO regions, while the motor neuron population was calculated as the percentage of ChAT^+^ area in the whole NMO region.

Neural progenitors were quantified based on immunofluorescence signals of Ki67 and SOX1 in the neural NMO compartment. In ilastik, images corresponding to each biomarker and to DAPI were segmented, and the exported masks and *.npy arrays were processed in Python. SOX1 and Ki67 masks were filtered by the DAPI mask and the neural region mask. In the neural region, the percentage of SOX1^+^ cells was calculated as the area of the SOX1^+^ signal divided by the area of the DAPI^+^ signal. The percentage of Ki67^+^ cells was calculated similarly.

Muscle markers were quantified based on whole NMO sections labeled for MyoD1, and in sections labeled for PAX7 and Ki67, both counterstained with DAPI. Ilastik was again used to generate masks for each nuclear biomarker and for DAPI. In Python, MyoD1 and PAX7 masks were filtered by the DAPI mask and the muscle region mask. Expression of MyoD1^+^ and PAX7^+^ cell populations in the muscle region was calculated as the area of the respective signal divided by the area of the DAPI^+^ signal. Similarly, the percentage of Ki67^+^ cell population was calculated in the muscle region and in the whole NMO.

In all Python pipelines, results were exported as *.xlsx files, along with confirmation images, for validation and downstream data analysis.

SOX10^+^/α‐ΒΤΧ^+^ and S100β^+^/α‐ΒΤΧ^+^ NMJ‐like structures were quantified based on spinning disc confocal immunofluorescence images. 20x renderings from these images, corresponding to NMO muscle regions (TTNGFP^+^ areas), were used to manually count the number of areas with overlapping/co‐localized expression/signal of each set of biomarkers, using the multi‐point tool in FiJi ImageJ.

Finally, SV2^+^ and SV2^+^/α‐ΒΤΧ^+^ regions were quantified based on immunofluorescence images acquired with a confocal microscope. 63× renderings corresponding to the muscle region (TTNGFP^+^ areas) were used to manually count areas exhibiting overlapping/co‐localized presynaptic and postsynaptic marker signal using the multi‐point tool in FiJi ImageJ.

In all image analyses, only images of NMO sections with neural or muscle tissue area <65% (or >35%) of the whole NMO area are included, to ensure that only sections corresponding to the major, inner volume of the NMO will be evaluated, thus excluding unrepresentative/outer layers.

#### 4.5.4 Calculation of NMO Muscle Region Proportion

The NMO muscle tissue size was calculated based on immunofluorescence image data, using whole NMO sections, labeled for muscle‐ and neural‐specific markers (e.g., panels including (i) Fast MyHC‐TUBB3, (ii) SMI32‐TTNGFP). Three independently labeled sections per NMO, per independent differentiation, were used to measure the whole organoid area and the NMO muscle area in each section, using the free‐hand selection tool in FiJi ImageJ. Before calculating the muscle area per NMO, the filtering threshold from before (Section 4.5.3) was again applied to confirm that only sections that correspond to the main volume of the NMO (65%–35% neural‐muscle or muscle‐neural tissue proportion) were included in the analysis. Then, the mean value of the whole organoid and muscle areas of three sections per NMO was calculated to obtain the *% of muscle tissue area* in each NMO.

### 4.6 Transmission Electron Microscopy

NMOs were fixed at room temperature for 2 h in a solution containing 2% formaldehyde (Electron Microscopy Sciences) and 2.5% glutaraldehyde (Sigma–Aldrich) in 0.1 M phosphate buffer, pH 7.2. Subsequently, the organoids were dissected into three smaller pieces (under a stereoscope), and sections that contained both neural and muscle tissues (where the NMJ‐like structures are primarily located) were then fixed for an additional 24 h at 4°C in freshly prepared fixative. Samples were then rinsed and treated with 1% aqueous osmium tetroxide (Electron Microscopy Sciences) for 2 h at room temperature. Following extensive washing with Milli‐Q water, en bloc staining was performed by incubating the samples overnight at 4°C in 0.5% aqueous uranyl acetate (Serva). After washing, samples were dehydrated through a graded acetone (Electron Microscopy Sciences) series (30%, 50%, 70%, 90%, and 2 × 100%) and infiltrated with epoxy resin (PolyBed 812, Polysciences) using acetone:resin mixtures (1:1 and 1:2). Final infiltration with 100% resin was conducted overnight on a rotator. Polymerization was carried out for 48 h at 60°C.

Ultrathin sections (80 nm) were cut using a Diatome Ultra 45° diamond knife on a Leica Ultracut S ultramicrotome. Sections were collected on Formvar‐carbon‐coated grids and post‐stained with uranyl acetate (Serva) and lead citrate (Leica) using a Leica EM AC20 automatic contrast system. Imaging was performed on a JEOL JEM‐2100 Plus transmission electron microscope (JEOL Ltd., Japan) equipped with the Xarosa CMOS camera (EMSIS GmbH).

### 4.7 Functional Characterization of NMOs

#### 4.7.1 NMO Contraction Analysis

Contraction of NMOs (day 60, 75) was recorded using a confocal microscope (Leica SP8), in incubation mode (37°C and 5% CO_2_). Two hours before the recordings, EPS‐NMOs or non‐paced control NMOs were collected and fresh N2B27 media was administered. One NMO per recording was placed in an empty well of the 12‐well plate with a minimum amount of N2B27 media (∼50 µL) in order to immobilize the sample and avoid drifting. With the 10x objective, a brightfield image of the sample was acquired and used as a map to determine the location of the recording: either of the two outer edges of the NMO, where neural and muscle regions intercalate. Then, one 10‐min recording (6222 frames, 0.096 frames/s) of the chosen location was acquired using a 4.5× magnification, set such that roughly half of the frame is filled with the organoid. Live recordings from at least four NMOs from either control or EPS‐trained organoids, from each independent differentiation and each independent genetic background were acquired.

To record the contractile activity of NMOs in response to pharmacological stimulation, fresh, warm N2B27 supplemented with glutamate (25µΜ) was added in the well of the 12‐well plate, already containing one NMO. Media were then gradually aspirated so that the NMO will “sink” in the middle of the well, with a minimum amount of N2B27 supplemented with glutamate (∼50 µL) in order to immobilize the sample and avoid drifting, as before. Live recordings from at least 5 NMOs from either non‐paced, control, or EPS‐trained organoids, from each independent differentiation, were acquired.

To analyze the spontaneous and the glutamate‐induced contractile activity of NMOs from the above dataset, videos were processed in Python, upon developing a custom two‐stage semi‐automated analysis pipeline.

##### 4.7.1.1 Signal Extraction

During this stage, the movement of the organoid border over time is extracted as a *Time Series* signal. First, an intensity threshold, determined automatically using Otsu thresholding on the first video frame, is set to obtain a binary segmentation of the video. If necessary, this threshold is then adjusted manually by the user before a binary segmentation of each video frame is generated. This is followed by a few morphological operations to obtain the final binary mask, from which the organoid border is extracted. Next, the length of the organoid border is obtained for computing the minimum border length over the whole video, to ensure that the same border points are acquired throughout the recording.

Given that in each contraction recording, organoids have different orientations, a common point of reference for all organoid borders is required to measure their movement over time. To this end, each video frame is rotated by an angle (*θ*) either clockwise or counter‐clockwise, such that the organoid border is always aligned to the *y*‐axis. The rotated image is then cropped using the previously computed minimum border length. The resulting images include only the minimum border region, meaning the border region is consistently visible across all frames. Finally, the border is split into 10 sub‐regions to ensure that localized twitches and subtle contractions will be included, and the average shift of the organoid border with respect to frame zero (i.e., displacement of the organoid upon contraction) for each sub‐region is calculated. This results in the extraction of 10 signals from each video, with length ***n*** equal to the number of frames in the video (6222 frames, 0.096 frames s^−1^), visualized as *Time Series* plots.

##### 4.7.1.2 Signal Analysis

First, time series data undergo a pre‐processing step, including polynomial interpolation of missing values (e.g., missing pixels in some border regions), conversion of time series units to physical quantities to match confocal settings (e.g., 1 pixel = 1.013 µm), noise reduction by smoothing, and data de‐trending as a means to remove potential organoid drifting from the evaluation of the contractile activity. Contraction features are then extracted using specific features of interest from the Python package *tsfresh (Time Series FeatuRe extraction on the basis of Scalable Hypothesis test)* [92]:

###### 4.7.1.2.1 Mean Displacement

It is defined as the average absolute displacement of the time series signal, given by the following equation:

(1) $$D_{n}=\frac{1}{n\Delta t}\sum_{i=1}^{n}\left|y_{i}\right|$$

where *y_i_* represents the displacement of the organoid upon contraction with respect to frame zero, *n* is the length of the time series, i.e., the number of frames in the video, and Δ*t*  =  0.096 *s* is the time resolution of the signal. This feature summarizes the overall displacement of the organoid border during the 10‐min recording, giving an indication of how much the organoid moves upon contraction.

###### 4.7.1.2.2 Contraction Power

To extract the absolute energy of our *Time series* signal, *y(t)*, which is the displacement (*y*‐axis in *Time series* plots) of the NMO border over time, we used the Python package ts‐fresh, in which the absolute energy of a signal is defined as the sum of the squared values of a signal, according to the following equation:

(2) $$E_{n}=\sum_{i=1}^{n}y_{i}^{2}\;$$

As this is often used as a proxy of the overall magnitude of the signal across its duration, it was considered a suitable model for the analysis of our *Time series* signal, *y(t)*, thereby defining *E_n_* as the area under the curve of the squared time series. However, this equation does not account for signal duration. To address this, we incorporated the time resolution of each signal into the analysis by dividing the absolute energy by the duration of the signal (i.e., the number of bins*, n*, multiplied by the time resolution for a discrete signal*, Δt*). We define this as *Contraction power*, computed according to the following equation:

(3) $$P_{n}=\frac{1}{n\Delta t}\;\sum_{i=1}^{n}y_{i}^{2}$$

In this study, the time resolution of the signal is *Δt = 0.096 s*, corresponding to the sampling interval of the time series. Consequently, the units of the contraction power are µm^2^ s^−1^. Since the absolute energy represents the area under the squared signal, the contraction power represents the average value of the squared signal over time, acting as an indicator of the average intensity of the signal and, thereby, an indirect measure of the NMO contraction strength.

###### 4.7.1.2.3 % of Count Above Threshold

This feature describes the percentage of values in the time series (*y(t)*) that are higher than the given threshold (*th*) below which movements are considered noise. Empirically, this threshold is set at 0.5 µm, in order to capture both strong contractions with prominent displacement and more subtle movements of the organoids, but could be adjusted by the user if deemed necessary.

Finally, features are extracted for each contraction video as raw data in *.xlsx files for downstream analysis.

#### 4.7.2 Multi‐Electrode Array (MEA) Recordings and Analysis

For day 60 MEA recordings, NMOs were carefully transferred to a 6‐well MEA plate, with 64 PEDOT electrodes per well (Cytoview MEA 6‐Black, Axion BioSystems), containing 1 mL of NB medium supplemented with 25 µM of glutamate. Excess of glutamate‐supplemented NM medium was then removed so that only a very thin layer remained on the recording area of each well, to allow for NMO attachment and viability during the recordings. If necessary, the position of the organoid was adjusted carefully so that it sits directly on the recording area of each well. The MEA plates were then transferred to the Maestro Pro MEA system (Axion Biosystems), which was already set up in incubator mode (37°C, and 5% CO_2_). 5‐min recordings of the NMO activity upon glutamate stimulation were acquired using AxIS Navigator software using the manufacturer's configuration for neural spontaneous activity. Data were analyzed using the manufacturer's standalone Neural Metric tool (Axion Biosystems). The spike detection threshold was set at 5 standard deviations, and electrodes that detected at least 5 spikes/min were classified as active. To evaluate bursting activity, an Inter‐Spike Interval (ISI) threshold requiring a minimum of 5 spikes with a maximum ISI of 100 ms was used. Synchronous activity/network burst was identified using an ISI threshold requiring at least 30 spikes of 100 ms maximum ISI, with a minimum of 10% of active electrodes. Firing synchrony was then automatically calculated as the area under the normalized synchrony cross‐correlogram for a time window of 20 ms. Data were then exported as *.csv files and processed for further analysis.

#### 4.7.3 Calcium Imaging of NMOs

On day 60, NMOs were collected under a stereomicroscope and embedded (one NMO per well) in a 96‐well PhenoPlate (PerkinElmer), coated with Matrigel (Gibco, Thermo Fisher Scientific). NMOs were maintained for four days, with half of the medium carefully replaced every other day. Such acute adherence of NMOs on the imaging plate enabled live calcium imaging of intact NMOs, at single‐cell resolution and with minimal sample movement during calcium indicator and/or glutamate injection [93]. On the day of the experiment (i.e., day 64 or day 4 post‐embedding), NMOs were incubated at 37°C with 4 µM of the cell‐permeant calcium indicator Fluo‐8 AM (abcam). After a 30‐min incubation, cells were washed once with fresh, warm culture medium (N2B27 without phenol red) and left to recover for 5 min in the incubator prior to calcium imaging recordings. Fluorescence time‐lapse recordings of calcium activity were acquired with a spinning disc confocal microscope (Opera Phenix Plus, PerkinElmer), in incubation mode (37°C, 5% CO_2_), using a custom automated acquisition pipeline. Background calcium transients were recorded for 1 min at 1 frame/s, followed by the addition of glutamate (25 µM; Sigma–Aldrich). Calcium transients following stimulation were recorded for 1.5 min at 10 frames/s.

Analysis of the calcium activity was performed using ImageJ (Fiji version 2.16) and custom Python and R routines. For the purpose of this experiment, only fields where cells appeared to be active at baseline were used. ROIs were detected manually, with neurons and muscle cells distinguished by morphology, including a background ROI for signal normalization. Fluorescence signals were quantified per ROI as relative changes in Fluo‐8 signal intensity for each frame, using the following equation:

(4) $$\Delta F/F_{0}=\frac{F-F_{0}}{F_{0}}\;$$

where *F*_0_ represents the average fluorescence value of the baseline recorded before glutamate stimulation. The maximum variation of fluorescence intensity (*Max ΔF/F_0_*), the delay in the response (*Time‐to‐peak (sec)*), and the decay of the calcium signal (*Time from Max ΔF/F_0_ to F_0_)* for each individual ROI were plotted and analyzed in Prism (GraphPad).

### 4.8 Reverse Transcription Quantitative PCR Analysis (qRT‐PCR)

Total RNA was isolated using the Direct‐zol RNA Miniprep Plus Kit (ZYMO RESEARCH), following the manufacturer's guidelines. Samples were stored at −80°C until ready for analysis. cDNA synthesis was performed with Superscript III system (Life Technologies), using random primers, and was amplified using Platinum SYBR‐Green (Invitrogen). PCR primers were designed using NCBI Primer‐Blast software, using exon‐spanning junctions (Table S6). For qPCR, the QuantStudio 6 Flex Real‐Time PCR system (Applied Biosystems) was used. Expression values for each gene were normalized against GAPDH, using the delta CT method. The mean expression value and standard deviation of two technical replicates per primer per sample were calculated in Microsoft Excel and plotted and analyzed in Prism 10. Standard deviations were calculated and plotted using GraphPad Prism 9. To obtain the gene expression ratios, the mean expression value for each gene was used to determine the fold‐change to day 60 control NMOs, in two independent experiments.

### 4.9 Characterization of NMO Mechanobiological Properties

#### 4.9.1 Video Acquisition

NMOs were placed in the IonOptix stimulation chambers connected to the pulse generator (as described earlier). Contractions were induced upon EPS‐stimulation of 1 Hz, 10 V, 10 ms. Time‐lapse brightfield videos were recorded using a Leica DMi1 Stand microscope with FLEXACAM C1 and accompanying software (Enersight, Leica Microsystems), during EPS‐induced contractions and subsequent relaxation time. All videos are of the same length (60 s, electrical pulses are delivered after the first 10 s) and were acquired under identical imaging conditions, before being stored for further analysis.

#### 4.9.2 Image Processing and Strain Analysis

Analysis was performed for videos exhibiting the full dark region of the muscular region using Fiji. Organoid boundaries were identified using adaptive thresholding and morphological filtering. The Particle Analyzer was used to extract the area. Python (PyCharm 2022.2.2) with OpenCV and NumPy was further used to plot A/A0 and extract peak strain as an index of contractile strength of the neuromuscular tissue. Relaxation dynamics (*tau*) were obtained by fitting the relaxation phase to an exponential decay model (Figure S12). The relaxation time constant *tau* was interpreted as a measure of tissue stiffness and viscoelastic recovery. Lower *tau* values correspond to faster relaxation and mechanically stiffer organoids.

#### 4.9.3 Data Aggregation and Statistical Analysis

Strain parameters (peak strain, relaxation rate constants, residual strain) were summarized per organoid. Results across groups were aggregated over multiple experiments into a *.csv file for downstream analysis.

### 4.10 Statistical Analysis

Statistical significance was tested in GraphPad Prism 10. The number of biological and technical replicates, along with statistical tests used, are indicated in the figure legends. Significant differences are indicated as ^*^ *p* ≤ 0.05; ^**^ *p* ≤ 0.01; ^***^ *p* ≤ 0.001; ^****^ *p* ≤ 0.0001. Data are provided in a single Data Source File.

## Author Contributions

M.G. and C.M.‐M. conceived the project and the experimental design. M.G. supervised the work, thoroughly reviewed and edited the manuscript, and provided critical feedback on data interpretation. C.‐M.M. designed and performed the experiments, collected all data and performed data analysis and interpretation, prepared the figures, and wrote the manuscript. I.A.M. established the pipelines for quantification of imaging data. I.A.E. analyzed the bulk RNA sequencing data and prepared the respective figures. D.C, C.B, and I.M. established the automated analysis pipeline for organoid contraction properties and size quantification and M.P. supervised this work and provided feedback. A.N. generated and provided KOLF NMPs. I.L. generated and provided WTC^mTTNGFP^ NMPs. E.K. developed the pipeline and analyzed the mechanobiological properties of NMOs. I.B. performed the calcium imaging assay. M.‐C.R. prepared the samples and acquired TEM data. All authors discussed the results and reviewed the manuscript.

## Conflicts of Interest

Mina Gouti has filed patents for the method of human neuromuscular organoid generation. The remaining authors declare no competing interests.

## Supporting information




**Supporting File**: advs76097‐sup‐0001‐SuppMat.docx.

**Supporting Figure**: advs76097‐sup‐0002‐SuppFigures.xlsx.

**Supporting Figure**: advs76097‐sup‐0003‐MainFigures.xlsx.

**Supporting Movie S1**: advs76097‐sup‐0004‐MovieS1.avi.

**Supporting Movie S2**: advs76097‐sup‐0005‐MovieS2.avi.

**Supporting Movie S3**: advs76097‐sup‐0006‐MovieS3.mp4.

**Supporting Movie S4**: advs76097‐sup‐0007‐MovieS4.mp4.

**Supporting Movie S5**: advs76097‐sup‐0008‐MovieS5.mp4.

**Supporting Movie S6**: advs76097‐sup‐0009‐MovieS6.mp4.

**Supporting Movie S7**: advs76097‐sup‐0010‐MovieS7.mp4.

**Supporting Movie S8**: advs76097‐sup‐0011‐MovieS8.mp4.

**Supporting Movie S9**: advs76097‐sup‐0012‐MovieS9.mp4.

**Supporting Movie S10**: advs76097‐sup‐0013‐MovieS10.mp4.

**Supporting Movie S11**: advs76097‐sup‐0014‐MovieS11.mp4.

**Supporting Movie S12**: advs76097‐sup‐0015‐MovieS12.mp4.

**Supporting Movie S13**: advs76097‐sup‐0016‐MovieS13.mp4.

**Supporting Movie S14**: advs76097‐sup‐0017‐MovieS14.mp4.

**Supporting Movie S15**: advs76097‐sup‐0018‐MovieS15.mp4.

**Supporting Movie S16**: advs76097‐sup‐0019‐MovieS16.mp4.

**Supporting Movie S17**: advs76097‐sup‐0020‐MovieS17.mp4.

**Supporting Movie S18**: advs76097‐sup‐0021‐MovieS18.mp4.

**Supporting Movie S19**: advs76097‐sup‐0022‐MovieS19.mp4.

**Supporting Movie S20**: advs76097‐sup‐0023‐MovieS20.mp4.

**Supporting Movie S21**: advs76097‐sup‐0024‐MovieS21.mp4.

**Supporting Movie S22**: advs76097‐sup‐0025‐MovieS22.mp4.

**Supporting Movie S23**: advs76097‐sup‐0026‐MovieS23.mp4.

**Supporting Movie S24**: advs76097‐sup‐0027‐MovieS24.mp4.

**Supporting Movie S25**: advs76097‐sup‐0028‐MovieS25.mp4.

**Supporting Movie S26**: advs76097‐sup‐0029‐MovieS26.mp4.

**Supporting Movie S27**: advs76097‐sup‐0030‐MovieS27.mp4.

**Supporting Movie S28**: advs76097‐sup‐0031‐MovieS28.mp4.

## Data Availability

The data that support the findings of this study are available from the corresponding authors upon reasonable request. All analyses of sequencing data were executed on a SLURM‐managed HPC cluster (Max Cluster) at MDC‐Berlin using GNU parallel to parallelize QC, alignment, and counting across samples. Software versions and links to reference files (genome build and GTF) are provided in Table S3 and command‐line arguments and analysis scripts are available on GitHub (https://github.com/Gouti‐Lab/EPS‐Project). Image analysis was carried out using FiJi, iLastik and python scripts. Code is available on GitHub (https://github.com/Gouti‐Lab/EPS‐Project). The code used for size analysis and spontaneous contraction characterization in Python is available at https://github.com/HelmholtzAI‐Consultants‐Munich/NMOs‐Contraction. The programme for quantification of mechanobiological properties is available at https://github.com/enricoklotzsch/organoid_stress_relax/.
