# Supplementary material for: Enhancing Maturation of Human Neuromuscular Organoids via Electrical Stimulation
Source: Adv Sci (Weinh). 2026 Jun 22;13(50):e22762. doi: 10.1002/advs.202522762 (PMC13337066; doi:10.1002/advs.202522762)
Supplement: Supplementary file 1 [file ADVS-13-e22762-s015.docx]

**Supporting Information**

**Enhancing Maturation of Human Neuromuscular Organoids via Electrical Stimulation.**

*Chrysanthi-Maria Moysidou,^*^ Inês Afonso Martins, Ismail Amr El-Shimy, Iacopo Bicci, Donatella Cea, Christina Bukas, Isra Mekki, Mara-Camelia Rusu, Aylin Nebol, Ines Lahmann, Marie Piraud, Enrico Klotzsch, Mina Gouti^*^*

**Supplementary Figure 1: Supporting information for Figure 1d, related to WTC^mTTNGFP^ NMO Size. a** Representative brightfield images of non-paced control NMOs and EPS-NMOs between day 30-40 (Early-stage NMOs), at different timepoints. Scale bar 1 mm. **b** Representative brightfield images of non-paced control NMOs and EPS-NMOs between day 50-60 (Late-stage NMOs), at different timepoints. Scale bar 1 mm. **c** Quantification of NMO size, calculated using brightfield images and expressed as Area (mm^2^). The mean ± SD is shown for each experimental group (control in dots, EPS- NMOs in triangle). Each datapoint represents one NMO. Data from *N=3* independent experiments are analyzed by unpaired t-test with Welch correction (control: day 30 *n=159*, day 40 *n=170*, day 60 *n=98* NMOs; Early-stage: day 30: *n=153*, day 40: *n=109*, day 60 *n=69* NMOs; Late-stage: day 30 *n=166,* day 60 *n=72*, day 75 *n= 41* NMOs; **P ≤ 0.05; **P ≤ 0.01; ***P ≤ 0.001; ****P ≤ 0.0001)*.

**Supplementary Figure 2:** Evaluation of the EPS training effects on apoptosis levels in WTC^mTTNGFP^ NMOs.

**a** Representative high-content, high-resolution images of whole-NMO sections of control, Early-stage and Late-stage EPS-NMOs, on day 40 and 60 (on the respective days EPS stops in both experimental groups), immunofluorescently labelled for the SMI32 neurofilament marker (in red), cleaved Caspase-3 protein (cCas-3; in yellow), and counterstained for DAPI. Titin protein (TTNGFP in green) is inherently expressed by NMOs. Scale bar 200 μm. **b** Quantification of cCas-3 apoptotic levels in neural and muscle NMO regions, based on immunofluorescence image data in a. Each datapoint represents one NMO. Data from *N=3* independent experiments are analyzed by unpaired t-test with Welch correction (control: day 40 *n=12*, day 60 *n=11* NMOs; Early-stage: day 40: *n=12-14* NMOs; Late-stage: day 60 *n=15* NMOs; **P ≤ 0.05; **P ≤ 0.01; ***P ≤ 0.001; ****P ≤ 0.0001)*. **c** Representative high-content, high-resolution images of whole-NMO sections of control, Early-stage and Late-stage EPS-NMOs, on day 40 and 60 labelled for TUNEL (in yellow) and counterstained for DAPI (in blue). Titin protein (TTNGFP in gray) is inherently expressed by NMOs. Scale bar 200 μm. **d** Quantification of TUNEL^+^ apoptotic cells in neural and muscle NMO regions, based on images in c. Each datapoint represents one NMO. Data from *N=2* independent experiments are analyzed by unpaired t-test with Welch correction (control: day 40 *n=8*, day 60 *n=7* NMOs; Early-stage: day 40: *n=7* NMOs; Late-stage: day 60 *n=8* NMOs; **P ≤ 0.05; **P ≤ 0.01; ***P ≤ 0.001; ****P ≤ 0.0001)*.

**Supplementary Figure 3:** Additional data on NMJ-like structure features related to Figure 1, based on 63x confocal micrographs**. a** Confocal images of immunofluorescently labelled sections on WTC^mTTNGFP^ NMOs for neuronal (; TUBB3, in yellow), muscle (Fast MyHC, in magenta) and NMJ biomarkers (α-ΒΤΧ in cyan), counterstained for DAPI (in grey). Representative 63x fields of view shown in Figure 1e, (scale bar 50 μm), along with respective magnified renderings (bottom panel; scale bar 20μm), offering a close-up view in the NMO muscle fiber organization and co-localization with NMJ and neuronal markers. **b** Quantification analysis of NMJ>2μm features. The mean ± SD of *N=3* independent experiments is shown for each experimental group. Each datapoint represents one 63x micrograph (control: day 40 *n= 37* from 11 NMOs, day 60 *n=73* from 13 NMOs, day 75 *n=78* from 13 NMOs; Early-stage NMOs, stable: day 40 *n=78* from 13 NMOs, day 60 *n= 79* from 13 NMOs; Late-stage NMOs: day 60 *n=74* from 12 NMOs, day 75 *n= 85* micrographs from 15 NMOs;). Data are analyzed by unpaired t-test with Welch correction (**P ≤ 0.05; **P ≤ 0.01; ***P ≤ 0.001; ****P ≤ 0.0001)*.

**Supplementary Figure 4:** Supporting information for Figure 2b, related to EPS-effects on WTC^mTTNGFP^ NMO size **a** Representative brightfield images of non-paced control NMOs and EPS-NMOs, chronically trained between day 30-60, at different timepoints. Scale bar 1 mm. **b** Plots illustrating the growth of both non-paced control NMOs and EPS-NMOs over time, calculated using brightfield images and expressed as Area (mm^2^). The mean ± SD is shown for each experimental group. Each datapoint represents one NMO. Data from *N=3* independent experiments are analyzed by unpaired t-test with Welch correction (control: day 30 *n=159*, day 40 *n=170*, day 60 *n=98* NMOs; Chronic EPS: stable day 30: *n=144*, day 60 *n=83,* day 75: *n=35* NMOs; increasing: day 30 *n=166,* day 60 *n=77*, day 75 *n= 38* NMOs; **P ≤ 0.05; **P ≤ 0.01; ***P ≤ 0.001; ****P ≤ 0.0001)*.

**Supplementary Figure 5:** Supporting information for Figure 2, related to EPS-effects on WTC^mTTNGFP^ NMO apoptosis levels **a** Representative high-content, high-resolution images of immunofluorescently labelled whole-NMO sections of non-paced control and EPS-NMO samples on day 60, when EPS training stops. Samples were labelled for the SMI32 neurofilament marker (in red), cleaved Caspase-3 protein (c-Cas-3; in yellow), and counterstained for DAPI (in blue). Titin protein (TTNGFP in green) is inherently expressed by NMOs. Scale bar 200 μm. **b** Quantification of day 60 cCas-3 apoptotic levels in neural and muscle regions of NMOs, based on immunofluorescence imaging data in c. Each datapoint represents one NMO. Data from *N=3* independent experiments are analyzed by unpaired t-test with Welch correction (control: *n=11* NMOs; Chronic EPS: stable *n=14* NMOs, increasing *n=14* NMOs; **P ≤ 0.05; **P ≤ 0.01; ***P ≤ 0.001; ****P ≤ 0.0001)*. **c** Representative high-content, high-resolution images of whole-NMO sections of non-paced control and EPS-NMO samples on day 60 labelled for TUNEL (in yellow) and counterstained for DAPI (in blue). Titin protein (TTNGFP in gray) is inherently expressed by NMOs. Scale bar 200 μm. **d** Quantification of TUNEL^+^ apoptotic cells in neural and muscle NMO regions, based on image data in c. Each datapoint represents one NMO. Data from *N=2* independent experiments are analyzed by unpaired t-test with Welch correction (control: *n=7* NMOs; Chronic EPS: stable *n=8* NMOs, increasing *n=8* NMOs; **P ≤ 0.05; **P ≤ 0.01; ***P ≤ 0.001; ****P ≤ 0.0001)*.

**Supplementary Figure 6:** Transmission Electron Microscopy (TEM) of day 60 WTC^mTTNGFP^ NMOs. **a** Representative ΤΕΜ micrographs of the neural (left) and muscle (right) compartments in an EPS-NMO trained under stable parameters. The neuronal region exhibits densely packed and aligned axons, seen here in cross-section (annotated examples in dark blue), while neuronal cells also display the typical ultra-structure (three annotated examples in light blue, orange and yellow). Fibres in NMO muscle area exhibit typical sarcomeric features, including distinct Z lines (Z) and M bands (M), as well as characteristic actomyosin filaments. *Scale bar 1 μm.* Similar observations were made across all experimental groups. **b** Representative TEM renderings of a control, non-stimulated NMOs. The right panel corresponds to the annotated area (black dashed box) in the left panel. A neurite is observed at the intersection of three muscle fibres (rich in mitochondria), with one appearing to form an NMJ-like structure. Synaptic vesicles (SV) can be clearly observed in the presynaptic terminal, however, distinguishing the intercellular space (ICS) and confirming the presence of a basal lamina (BL) or secondary synaptic cleft formation (i.e., invagination) remains challenging in this sectioning plane. **c** Representative TEM renderings of an EPS-NMO trained under stable parameters. A close-up of the annotated area on the left (black dashed box) is shown in the right panel, revealing neurites positioned closely to the muscle cell. Characteristic features of NMJ formation at the synaptic cleft can be observed, including SVs at the presynaptic terminal of both neurites, close to the muscle cell, which in turn exhibits postsynaptic densities (orange arrow head). At the ICS, the BL (black arrow) is clearly visible, along with the secondary synaptic cleft (blue arrow head). **d** Representative TEM renderings of an EPS-NMO trained under increasing parameters. A close-up of the annotated area on the left (black dashed box) is shown in the right panel, depicting several neurites positioned closely to muscle cells. At the synaptic cleft, SVs at the presynaptic terminal, in close proximity to the muscle cell, can be observed. In turn, postsynaptic densities (orange arrow head) can be seen at the muscle cell plasma membrane, while signs of secondary synaptic cleft formation are also visible (blue arrow head). In this imaging plane, the presence of BL cannot be confirmed. Scale bar: left 1 μm, right 500 nm. **b-c:** S*cale bar: left 1 μm, right 500 nm.*

**Supplementary Figure 7**: Chronic EPS enhances NMJ formation in NMOs. a Immunofluorescence analysis of WTCmTTNGFP NMO sections stained for synaptic vesicle protein 2 (SV2, yellow), titin (inherent TTNGFP signal was further amplified with anti-GFP antibody, magenta), and AChR clusters (a-bungarotoxin; α-BTX, cyan), with nuclei counterstained with DAPI (grey). Representative whole organoid sections of day 60 NMOs are shown for each experimental group (scale bar: 250 μm), along with 63x fields of view of the respective NMOs (scale bar 50 μm). b Quantification of SV2+ and α-BTX^+^ NMJ-like structures, based on the 63x micrographs shown in a. Plots depict the absolute number of α-BTX^+^ NMJ-like structures per area (i.e., per 63x micrograph) and SV2+ and α-BTX^+^ NMJ-like structures per area on day 60 control, non-stimulated NMOs, and on Chronic EPS-NMOs, trained under stable or increasing parameters. The mean ± SD of N=1 independent experiment is shown for each experimental group. Each datapoint represents one 63x micrograph (n=6 micrographs per group, 2 NMOs per group). Data were analyzed by unpaired t-test with Welch correction (**P ≤ 0.05; **P ≤ 0.01; ***P ≤ 0.001; ****P ≤ 0.0001*).

**Supplementary Figure 8:** Supporting information for Figure 2c,d, related to EPS effects on NMJ-like structure features on day 75. **a** Representative confocal images (63x renderings) of immunofluorescently labelled sections of day 75 WTC^mTTNGFP^ non-paced control and EPS-NMOs, chronically trained until day 60. Samples were labelled for (tubulin-β-3; TUBB3, in yellow), muscle (Fast Myosin Heavy Chain; MyHC, in magenta) and NMJ biomarkers (a-bungarotoxin; α-ΒΤΧ in cyan), counterstained for DAPI (in grey). Scale bar 50 μm. Left panel corresponds to magnification renderings, enabling close-up view of NMJ, neuronal and muscle marker co-localisation. Scale bar 20 μm. **b** Quantification of NMJs>5 μm features over time, based on 63x micrographs in b. Plots show the absolute number per area (i.e., per 63x micrograph), innervation and average size of NMJs>5 μm for non-paced control NMOs and EPS-NMOs, trained under stable or increasing pulse parameters, on day 60 (last day of EPS training) and on day 75 (two-weeks post-EPS training). The mean ± SD of *N=3* independent experiments is shown for each experimental group. Each datapoint represents one 63x micrograph (Day 60: control: n=73 from 13 NMOs, Chronic EPS: stable: *n=84* from 14 NMOs; increasing: *n=84* from 14 NMOs; Day 75: control *n=34* from 12 NMOs, Chronic EPS: stable: *n=38* from 14 NMOs, increasing: *n=42* from 15 NMOs; Data are analyzed by unpaired t-test with Welch correction (**P ≤ 0.05; **P ≤ 0.01; ***P ≤ 0.001; ****P ≤ 0.0001)*. **c** Quantification of NMJs>2 μm features over time, based on 63x micrographs in b. Plots show the absolute number per area (i.e., per 63x micrograph), innervation and average size of NMJs>5 μm for non-paced control NMOs and EPS-NMOs, trained under stable or increasing pulse parameters, on day 60 (last day of EPS training) and on day 75 (two-weeks post-EPS training). The mean ± SD of *N=3* independent experiments is shown for each experimental group. Each datapoint represents one 63x micrograph (Day 60: control: *n= 73* from 13 NMOs, Chronic EPS: stable: *n=84* from 14 NMOs; increasing: *n=84* from 14 NMOs; Day 75: control *n=34* from 12 NMOs, Chronic EPS: stable: *n=38* from 14 NMOs, increasing: *n=42* from 15 NMOs; Data are analyzed by unpaired t-test with Welch correction (**P ≤ 0.05; **P ≤ 0.01; ***P ≤ 0.001; ****P ≤ 0.0001)*.

**Supplementary Figure 9:** Evaluation of WTC^mTTNGFP^ NMO spontaneous contraction on day 75. **a** Representative Time Series plots, illustrating the spontaneous contractile activity of non-paced control and chronically EPS-trained NMOs on day 75, during a 10-minute live recording. Plots in the right column are low-range scale insets of the corresponding plots in the left, illustrating more details about NMO contractile movements. **b** Quantitative analysis of the NMO spontaneous contractile activity on day 75, based on *Mean displacement (μm), Contraction power* (μm^2^ s^-1^) and *% of count above threshold* plots. Each datapoint represents the contractile activity of one NMO (i.e., one 10-minute live recording). Data from *N=3* independent experiments are analyzed by unpaired t-test with Welch correction (control: *n= 14* NMOs, Chronic EPS, stable: *n=15* NMOs, increasing: *n=15* NMOs; **P ≤ 0.05; **P ≤ 0.01; ***P ≤ 0.001; ****P ≤ 0.0001)*.

**Supplementary Figure 10:** Evaluation of chronic EPS training under stable pulse parameters in KOLF NMOs. **a** Representative brightfield images of day 60 non-paced control KOLF NMOs and EPS-NMOs, chronically trained between day 30-60. Scale bar 1 mm. **b** Quantification of KOLF NMO size, calculated using brightfield images and expressed as Area (mm^2^). The mean ± SD is shown for each experimental group (control in dots, EPS- NMOs in triangle). Each datapoint represents one NMO. Data from a representative experiment are analyzed by unpaired t-test with Welch correction (control: day 30 *n=29*, day 40 *n=11*, day 60 *n=10* NMOs; EPS-NMOs day 40: *n=13*, day 60 *n=12* NMOs; **P ≤ 0.05; **P ≤ 0.01; ***P ≤ 0.001; ****P ≤ 0.0001)*. **c** Confocal images of immunofluorescently labelled sections of day 60 KOLF non-paced control and EPS-NMOs, chronically trained until day 60. Samples were labelled for (tubulin-β-3;TUBB3, in yellow), muscle (Fast Myosin Heavy Chain; Fast MyHC, in magenta) and NMJ biomarkers (a-bungarotoxin; α-ΒΤΧ in cyan), counterstained for DAPI (in grey). Representative whole NMO images are shown for each experimental group, along with 63x fields of view of the respective NMOs. Scale bar: 250 μm for whole NMOs, 50 μm for 63x micrographs. **d** Quantification of NMJ-like structure features on day 60, based on 63x micrographs in c. Plots show the absolute number per area (i.e., per 63x micrograph), innervation and average size of NMJs>2μm and NMJs>5 μm for KOLF non-paced control NMOs and EPS-NMOs, trained under stable parameters on day 60 (last day of EPS training). The mean ± SD of *N=2* independent experiment is shown for each experimental group. Each datapoint represents one 63x micrograph (control: *n= 27* from 9 NMOs, EPS-NMOs: *n=30* from 10 NMOs; Data are analyzed by unpaired t-test with Welch correction (**P ≤ 0.05; **P ≤ 0.01; ***P ≤ 0.001; ****P ≤ 0.0001))*. **e** Representative Time Series plots and corresponding close-ups, illustrating the spontaneous contractile activity of KOLF non-paced control and chronically EPS-trained NMOs on day 60, during a 10-minute live recording. **f** Quantitative analysis of the KOLF NMO spontaneous contractile activity on day 60, based on *Contraction power* (μm^2^ s^-1^) and *% of count above threshold* features. Each datapoint represents one NMO (i.e., one 10-minute live recording). Data from *N=2* independent experiment are analyzed by unpaired t-test with Welch correction (control: *n= 9* NMOs, EPS-NMOs, stable: *n=9* NMOs; **P ≤ 0.05; **P ≤ 0.01; ***P ≤ 0.001; ****P ≤ 0.0001)*.

**Supplementary Figure 11:** Analysis of WTC^mTTNGFP^ NMO transcriptomic profile. **a** Dot plots illustrating the differentially expressed genes between day 60 non-paced control NMOs and chronic EPS-NMOs (stable pulse parameters in top panel; increasing pulse parameters in bottom panel), mapped to the relevant biological functions and cellular processes they regulate. Dot color indicates log₂ fold-change, and dot size represents the magnitude of statistical significance (*adjusted p-value <0.05*). **b** Normalized expression of DEGs (*adjusted p-value <0.05*) over time in non-paced control NMOs and EPS-NMOs. Top row plots depict the expression level trajectory of genes significantly enriched in both chronic EPS-NMOs, compared to non-paced NMOs. Bottom row panels illustrate the expression level trajectory of genes significantly enriched only in EPS-NMOs chronically trained under stable pulse parameters.

**Supplementary Figure 12:** Image analysis of the neural progenitor and proliferation marker expression levels in WTC^mTTNGFP^ non-paced control NMOs and in EPS-NMOs. **a** High-content, high-resolution images of whole-NMO sections, immunofluorescently labelled for the neural progenitor marker SOX1 (in red) and proliferative marker Ki67 (in yellow), counterstained for DAPI (in blue). Titin protein (TTNGFP in green) is inherently expressed by NMOs. Images are representative of EPS-trained NMOs on day 40 (Early-stage) and day 60 (Chronic EPS-NMOs, under stable or increasing pulse parameters), and of non-paced control NMOs on the respective timepoints. Scale bar 200 μm. **b** Quantitative analysis of the expression of SOX1^+^ cells and Ki67^+^ cells in the neural region of EPS-trained NMOs on day 40 (Early-stage) and day 60 (Chronic EPS-NMOs, stable or increasing), and of non-paced control NMOs on the respective timepoints, based on images in a. Each datapoint represents one NMO. Data from *N=3* independent experiments are analyzed by unpaired t-test with Welch correction (Day 40: control *n= 11-13* NMOs, Early-stage: *n=13-14* NMOs; Day 60: control *n=12-13* NMOs, Chronic EPS: stable *n=14* NMOs, increasing *n=14* NMOs; **P ≤ 0.05; **P ≤ 0.01; ***P ≤ 0.001; ****P ≤ 0.0001)*.

**Supplementary Figure 13:** Supporting information for Figure 3, related to EPS effects on WTC^mTTNGFP^ NMO neural tissue cell composition and organization. **a** Representative high-content, high-resolution images of whole NMO sections on day 40, illustrating the presence of glial fibrillary acidic protein positive cells (GFAP, in yellow) of non-paced control and Early-stage EPS-NMOs. Scale bar 200 μm. **b** Plot illustrating the expression of GFAP^+^ cells in the whole organoid of EPS-NMOs on day 40 (Early-stage) and day 60 (Chronic EPS, stable or increasing), and of non-paced control NMOs on the respective timepoints, based on images in Fig 4a. Each datapoint represents one NMO. Data from *N=3* independent experiments are analyzed by unpaired t-test with Welch correction (control: day 40 *n=9*, day 60 *n=11* NMOs; Early-stage: day 40 *n=11* NMOs; Chronic EPS, stable: day 60 *n=12* NMOs, increasing: day 60 *n=13* NMOs; *P ≤ 0.05; **P ≤ 0.01; ***P ≤ 0.001; ****P ≤ 0.0001). **c** Representative high-content, high-resolution images of whole NMO sections (left panel) on day 60, illustrating the presence of calcium-binding protein B positive Terminal Schwann cells (S100β, in yellow), in WTCmTTNGFP non-paced control and in EPS-NMOs. Images in the right panel are close-ups of annotated areas in the muscle NMO region (TTNGFP, pseudo-colored grey) of images in the left panel, revealing that S100β^+^ cells are present at very close proximity with NMJ-like structures (α-ΒΤΧ, in dark red). Scale bar: left panel 200 μm, right panel 50 μm. **d.** Quantification of α-ΒΤΧ^+^ and S100β^+^ NMJ-like sites in day 60 control, non-paced NMOs, and Chronic EPS-NMOs, based on immunofluorescence data in c. Data from *N=1* independent experiment are analyzed by unpaired t-test with Welch correction (*n=3* 20x field of view renderings from 3 NMOs per experimental group *P ≤ 0.05; **P ≤ 0.01; ***P ≤ 0.001;) **e** Representative high-content, high-resolution images of day 60 whole NMO sections (left panel), labelled for Transcription factor protein SOX10 (in yellow), and α-ΒΤΧ AChR clusters (in dark red), counterstained for DAPI (in blue). Images in the right panel are close-ups of annotated areas in the muscle NMO region (TTNGFP, pseudo-colored grey) of images in the left panel, revealing co-localisation of SOX10^+^ cells with α-ΒΤΧ^+^ NMJ-like structures. Scale bar: left panel 200 μm, right panel 50 μm. **f** Plots demonstrating the numbers of SOX10^+^ and α-ΒΤΧ^+^ NMJ-like structures, based on immunofluorescence data in e. Data from *N=1* independent experiment are analyzed by unpaired t-test with Welch correction (*n=3* 20x field of view renderings from 3 NMOs per experimental group *P ≤ 0.05; **P ≤ 0.01; ***P ≤ 0.001;)

**Supplementary Figure 14: a** Plots depicting the magnitude of Burst Frequency (Hz) and Number of Spikes participating per Burst during 5-minute MEA recordings of glutamate-induced (25μM) activity of control and EPS-NMOs on day 60. Each data point represents the number of NMOs that exhibited bursting events during recordings. Data from *N=2* independent experiments are analyzed by unpaired t-test with Welch correction (control: *n=3* NMOs; stable: *n=5* NMOs; increasing: *n=7* NMOs; **P ≤ 0.05; **P ≤ 0.01; ***P ≤ 0.001; ****P ≤ 0.0001*). **b** Representative normalised cross-correlograms used as a metric of synchronous NMO neural tissue activity during recordings in a. **c** Representative snapshots (left) of time-lapse recordings of glutamate-induced calcium-dependent neuronal activity, based on Fluo-8 AM indicator, of day 60 non-paced, and Chronic EPS-NMOs. The white line marks the border of the organoid under evaluation in each case. Right panel illustrates representative calcium traces (*ΔF/F_0_*) for corresponding ROIs in each snapshot in the left panel (also see Movies SX-SX). **d** Calcium transient analysis. Plots exhibit the magnitude of peak change in fluorescence intensity relative to baseline (*Max ΔF/F_0_*), and calcium kinetics, *Rise Time/Time Max ΔF/F_0_* (s)) and *Decay Time*/*Time from Max ΔF/F_0_ to F_0_* (s) of the glutamate-induced NMO response of all experimental groups. Data from *N=3* independent experiments are analyzed by unpaired t-test with Welch correction. Each dot represents an ROI trace, corresponding to the activity of one neuronal cell (*n=7-1*5 neuronal cell per NMOs; 5 NMOs per experimental group; **P ≤ 0.05; **P ≤ 0.01; ***P ≤ 0.001; ****P ≤ 0.0001*).

**Supplementary Figure 15:** Image analysis of muscle progenitor and proliferation marker expression levels in WTC^mTTNGFP^ non-paced control and EPS-NMOs, related to Figure 4b, and quantification of myotube and myofiber features, related to Figure 4d. **a** High-content, high-resolution images of whole-NMO sections, immunofluorescently labelled for the satellite-like cell marker PAX7 (in red) and proliferative marker Ki67 (in yellow), counterstained for DAPI (in blue). Titin protein (TTNGFP in green) is inherently expressed by NMOs. Images are representative of EPS-trained NMOs on day 40 (Early-stage) and day 60 (Chronic EPS, stable or increasing), and of control NMOs on the respective timepoints. Scale bar 200 μm. **b** Plot illustrating the expression of PAX7^+^ cells in the muscle region of EPS-NMOs on day 40 (Early-stage) and day 60 (Chronic EPS, stable or increasing), and of non-paced control NMOs on the respective timepoints. Each datapoint represents one NMO. Data from *N=3* independent experiments are analyzed by unpaired t-test with Welch correction (control: day 40 *n=12*, day 60 *n=12* NMOs; Early-stage: day 40 *n=12* NMOs; Chronic EPS, stable: day 60 *n=13* NMOs, increasing: day 60 *n=13* NMOs; **P ≤ 0.05; **P ≤ 0.01; ***P ≤ 0.001; ****P ≤ 0.0001*).

**Supplementary Figure 16:** Supporting information for Figure 4c, related to evaluation of WTC^mTTNGFP^ NMO muscle tissue properties. High-content, high-resolution images of whole-NMO sections, immunofluorescently labelled for the myogenin progenitor marker MyoD1 (in yellow) and counterstained for DAPI (in blue). Titin protein (TTNGFP; in green) is inherently expressed by NMOs. Images are representative of EPS-NMOs on day 40 (Early-stage) and day 60 (Chronic EPS, stable or increasing), and of non-paced control NMOs on the respective timepoints. Scale bar 200μm.

**Supplementary Figure 17:** Supporting information for Figure 4d, related to evaluation of WTC^mTTNGFP^ NMO muscle tissue properties over time (d40-d60) in WTC^mTTNGFP^ non-paced control NMOs and EPS-trained NMOs. **a** Additional data on quantification of myotube and myofiber features in Figure 4d. In the plot of Number of myotubes, each data point represents the mean number of myotubes per 63x micrograph, per NMO (day 40: control: *n=31* micrographs, 11 NMOs, Early-stage: *n= 39* micrographs, 13 NMOs; day 60: control: *n=36* micrographs, 13 NMOs, stable: *n=42* micrographs, 14 NMOs, increasing: *n=42* micrographs, 14 NMOs). In the plot for Myotube size, each datapoint represents one myotube (day 40: control: *n=295,* 11 NMOs, Early-stage: *n= 358*, 13 NMOs; day 60: control: *n=348*, 13 NMOs, stable: *n=373*, 14 NMOs, increasing: *n=342*, 14 NMOs). Data from *N=3* independent experiments are analyzed by unpaired t-test with Welch correction (day 60 *n=42* from 14 NMOs; **P ≤ 0.05; **P ≤ 0.01; ***P ≤ 0.001; ****P ≤ 0.0001*). **c** Plot illustrating the % of NMO area covered by muscle tissue (*% of muscle area*), based on quantification of the NMO region exhibiting positive signal for muscle-specific markers, including TTNGFP inherent signal seen in panel a here. Each dot represents the mean value per NMO from *N=3* independent experiments. At least three images per NMO were analysed (control: day 40 *n= 10* NMOs, day 60 *n=9* NMOs; Early-stage: day 40 *n=10* NMOs; Chronic EPS, stable: day 60 *n=11* NMOs, increasing: day 60 *n=11* NMOs;) Data are analyzed by unpaired t-test with Welch correction (**P ≤ 0.05; **P ≤ 0.01; ***P ≤ 0.001; ****P ≤ 0.0001*).

**Supplementary Figure 18:** Evaluation of gene expression related to NMJ and muscle tissue characterisation data in Figure 4. **A** Fold-change of the gene expression level ratio of *CHRNE* and *CHRNE* genes, coding for AChR epsilon (ε) and gamma (γ) subunits of NMJs, in day 60 control, non-paced and Chronic EPS-NMOs. **b** Plots illustrating fold-change in gene expression level ratio of myosin isoforms (*MYH3, MYH4, MYH2*), encoding for developmental and more mature fibers in day 60 control, non-paced and Chronic EPS-NMOs. Data from *N=2* independent experiments are analysed by unpaired t-test with Welch correction (**P ≤ 0.05; **P ≤ 0.01; ***P ≤ 0.001; ****P ≤ 0.0001*). At least 5 NMOs per group were used for RNA extractions, with *n=2* technical replicates per group used for calculating mean fold-changes ± SD, in a and b.

**Supplementary Figure 19:** **a** Representativr high-content, high-resolution micrographs of day 60 WTC^mTTNGFP^ NMO sections, immunofluorescently labelled for neurofilament (SMI32, in dark pink), titin (inherent TTNGFP signal was further boosted with anti-GFP antibody, in grey), and collagen IV (COL4A1, in yellow), counterstained for DAPI (in blue). Representative images are shown for each experimental group (top row), along with the corresponding image for COL4A1 (bottom row). Scale bar: 200 μm. **b** Quantification of collagen IV expression in whole NMO, neural, and muscle NMO area, based on micrographs in a. Plots depict the % of COL4A1^+^ area for each experimental group, expressed as mean ± SD of *N=2* independent experiments (control: *n=7* NMOs, Chronic EPS: stable: *n= 1*0 NMOs, increasing *n= 7* NMOs). Data were analyzed by unpaired t-test with Welch correction (*P ≤ 0.05; **P ≤ 0.01; ***P ≤ 0.001; ****P ≤ 0.0001).

**Supplementary Figure 20:** **a** Representativr high-content, high-resolution micrographs of day 60 WTC^mTTNGFP^ NMO sections, immunofluorescently labelled for neurofilament (SMI32, in dark red), titin (inherent TTNGFP signal was further boosted with anti-GFP antibody, in grey), and collagen V (*COL5A3*, in yellow), counterstained for DAPI (in blue). Representative images are shown for each experimental group (top row), along with the corresponding image for COL5A3 (bottom row). Scale bar: 200 μm. **b** Quantification of collagen V expression in whole NMO, neural, and muscle NMO area, based on micrographs in a. Plots depict the % of COL5A3^+^ area for each experimental group, expressed as mean ± SD of *N=2* independent experiments (control: *n=9* NMOs, Chronic EPS: stable: *n= 1*0 NMOs, increasing *n= 9* NMOs). Data were analyzed by unpaired t-test with Welch correction (*P ≤ 0.05; **P ≤ 0.01; ***P ≤ 0.001; ****P ≤ 0.0001).

**Supplementary Figure 21:** Supporting information for Figure 4e, related to evaluation of WTC^mTTNGFP^ NMO mechanical properties and respective analysis pipeline. **a** Brightfield image of an NMO, representative of a frame from one-minute live recordings of electrical stimulation-induced contractions. **b** Representative mask of the image in a, used for thresholding the muscle (dark region) of the NMO (*A*). **c** Plot illustrating the changes of muscle area (*A*) over time. Stimulation of the samples starts at 10 s and resumes until the end of the recording (60 s). Strain is calculated upon measuring the muscle area (*A*), normalized to the area in frame 0 (*A_0_-1*). *A* value of 0.01 represents a change in area of 1%. The maximum peak strain is calculated from the maximum and minimum of the first peak. **d** The plot illustrates the final step of the analysis pipeline for calculating the relaxation time constant *tau*. Individual curves from c are overlayed and fitted with an exponential decay model (shown in the plot), allowing for extraction of *tau*.

**Supplementary Tables**

**Table S1. hiPSC lines**

| Cell Line | Product ID  (hPSCreg ID) | Source | Sampling age (years) | Sex | Ref |
| --- | --- | --- | --- | --- | --- |
| WTC-mTTNGFP | AICS-0048-039  (UCSFi001-A-27) | Allen Cell Collection | 30-34 | male | ^[1]^ |
| KOLF2.1J from HPSI0114i-kolf_2 | JIPSC001000  (WTSIi018-B) | The Jackson Laboratory | 55-59 | male | ^[2]^ |

**Table S2. Primary and secondary antibodies for immunofluorescence staining**

| Antibody | Manufacturer/Source | Identifier | Dilution |
| --- | --- | --- | --- |
| Goat anti-choline acetyltransferase (ChAT) | Millipore | AB144P | 1:200 |
| Rabbit anti-cleaved Caspase-3 | Cell Signaling | 9661S | 1:1000 |
| Mouse anti-neurofilament H (SMI32) | Biolegend | 801701 | 1:1000 |
| Mouse anti-Myosin Skeletal Fast (Fast Myosin Heavy Chain; MYH1/MYH2) | Sigma Aldrich | M4276 | 1:500 |
| Rabbit anti-beta Tubulin 3/ Tuj1 (TUBB3) | Biozol | GTX129913 | 1:1000 |
| Mouse anti-Myogenic Differentiation 1 (MyoD1) | BD | 554130 | 1:50 |
| Mouse anti-Paired Box 7 (Pax7) | DSHB | PAX7-s | 1:50 |
| Mouse anti-SV2A | DSHB | SV2 | 1:500 |
| Goat anti-Sox1 | R&D | AF3369 | 1:500 |
| Rabbit anti-Ki67 | abcam | ab15580 | 1:501 |
| Rabbit anti-S100b | abcam | ab52642 | 1:500 |
| Rabbit anti-GFAP | Sigma Aldrich | SAB5600060 | 1:500 |
| Rabbit anti-COL4A1 | Abcam | ab6586 | 1:200 |
| Rabbit anti-COL5A3 | Proteintech | 30204-1-AP | 1:200 |
| Goat anti-SOX 10 | Biotechne | AF2864 | 1:1000 |
| TUNEL Assay Kit (Fluorescence, 594 nm) | Cell Signaling | #48513 |  |
| Alexa 647 Conjugate α bungarotoxin | Thermo Fisher | B35450 | 1:1000 |
| Donkey anti-mouse, Alexa Fluor 488 | Invitrogen | A21201 | 1:500 |
| Donkey anti-rabbit, Alexa Fluor 568 | Invitrogen | A10042 | 1:500 |
| Donkey anti-mouse, Alexa Fluor 568 | Invitrogen | A10037 | 1:500 |
| Donkey anti-mouse Alexa Fluor 647 | Invitrogen | A31571 | 1:500 |
| Donkey anti-goat, Alexa Fluor 647 | Invitrogen | A21447 | 1:500 |

**Table S3. Software used in bulk RNA sequencing data analysis**

| Software (version) | Citation |
| --- | --- |
| FastQC (v0.12.1) | ^[3]^ |
| MultiQC (v1.25.1) | ^[4]^ |
| HISAT2 (v2.2.1) | ^[5]^ |
| samtools (v1.22.1) | ^[6]^ |
| featureCounts (Subread v1.5.3-0) | ^[7]^ |
| R version 4.4.3 (2025-02-28) | R Core Team (2024). R: A Language and Environment for Statistical Computing. R Foundation for Statistical Computing, Vienna, Austria (https://www.R-project.org/). |
| RStudio 2024.12.1+563 "Kousa Dogwood" Release | Posit team (2024). RStudio: Integrated Development Environment for R. Posit Software, PBC, Boston, MA. URL http://www.posit.co/. |
| DESeq2 (v1.46.0) | ^[8]^ |
| fgsea (v1.32.4) | ^[9]^ |
| enrichR (v3.4) | Jawaid W (2025). enrichR: Provides an R Interface to 'Enrichr'. R package version 3.4, (https://CRAN.R-project.org/package=enrichR). |
| Reference file | **Link** |
| Human GRCh38 (GENCODE v49/Ensembl115 annotations) | https://ftp.ensembl.org/pub/release-115/fasta/homo_sapiens/dna/ |
| Homo sapiens GTF (Ensembl release-115 ) | https://ftp.ensembl.org/pub/release-115/gtf/homo_sapiens/Homo_sapiens.GRCh38.115.gtf.gz |

**Table S4. Reagents and consumables**

| Chemicals, Small molecules, reagents | Manufacturer/Source | Identifier |
| --- | --- | --- |
| 2-Mercaptoethanol | Gibco, Thermo Fisher Scientific | 31350010 |
| Accutase | Sigma-Aldrich | A6964 |
| B27 | Gibco, Thermo Fisher Scientific | 17504044 |
| BAMBANKER | Nippon | BB05 |
| BSA | VWR | A7906 |
| BSA for molecular biology | Sigma-Aldrich | A9418 |
| CHIR 99021 | Tocris | 4423/10 |
| Curare | Sigma-Aldrich | T2379 |
| DAPI | Thermo Fisher Scientific | D1306 |
| Direct-zol RNA Miniprep Plus Kit | ZYMO RESEARCH | R2071 |
| DMEM F12 | Gibco, Thermo Fisher Scientific | 21331046 |
| DPBS | Gibco, Thermo Fisher Scientific | 14190-169 |
| bFGF | in house |  |
| Geltrex LDEV-Free, hESC-Qualified | Gibco, Thermo Fisher Scientific | A1413302 |
| GlutaMAX Supplement | Gibco, Thermo Fisher Scientific | 35050-038 |
| HGF | Peprotech | 100-39H |
| IGF | Peprotech | 100-11 |
| Matrigel hESC­Qualified Matrix | Gibco, Thermo Fisher Scientific | 354277 |
| mTeSR | Stemcell Technologies | 85850 |
| N2 | Gibco, Thermo Fisher Scientific | 17502048 |
| NB | Gibco, Thermo Fisher Scientific | 21103049 |
| Paraformaldehyde | VWR | ALFAJ19943.K2 |
| Pen / Strep | Gibco, Thermo Fisher Scientific | 15140122 |
| Triton-X 100 | Sigma-Aldrich | T9284 |
| Trypan Blue | Gibco, Thermo Fisher Scientific | T10282 |
| Y-27632 (hydrochloride) | BioMol GmbH | Cay10005583-50 |
| Gelatin | Sigma-Aldrich | G1890 |
| Sucrose | Sigma-Aldrich | 84097 |
| Immu-Mount mounting medium | erpedia | 9990402 |
| 50 ml Falcon tubes | NeoLab | 352070 |
| 15 ml Centrifugation tubes | FAUST | TPP91015 |
| 60mm cell culture dishes | Sigma-Aldrich | CLS430166-500EA |
| 96-well U-bottom ultra-low attachment plates | Thermo Fisher Scientific | 174929 |
| Countess™ chamber | Thermo Fisher Scientific | C10228 |
| Cryovials | Thermo Fisher Scientific | 10418571 |
| Falcon® 6 Well TC-Treated Multiwell Cell Culture Plate | NeoLAb | 353046 |
| Microtome blades Type C35 | pfm medical | 207500003 |
| Superfrost Plus adhesion microsope slides | Epredia | J1800AMNZ |
| Cover slips, rectangular, 0.13-0.16mm thick | Th. Geyer | 7695031 |
| LoBind tubes 1.5 mL | VWR | 525-0133 |
| 96-well PhenoPlate | PerkinElmer | 6055302 |
| Fluo-8 AM | abcam | ab142773 |
| Glutamate | Sigma Aldrich | G1626-100G |
| CytoView MEA Plates | Axion BioSystems, USA | M384-tMEA-6B |
| Formaldehyde | Electron Microscopy Sciences | 15710 |
| Glutaraldehyde | Sigma-Aldrich | G5882-10 |
| Osmium tetroxide | Electron Microscopy Sciences | 19190 |
| Uranyl acetate | Serva | 77870 |
| Acetone | Electron Microscopy Sciences | 10015 |
| Resin | Polysciences | 8791 |
| Lead citrate | Leica | Ultrostain2 |
| SuperScript III Reverse Transcriptase | Life Technologies | 18080044 |
| Platinum SYBR Green | Invitrogen | 11744500 |

**Table S5. Equipment and software**

| Software | Manufacturer/Source |
| --- | --- |
| Microsoft Word 16.99.2 | Microsoft |
| Micosoft Excel 16.99.2 | Microsoft |
| GraphPad Prism 9 and 10 | GraphPad Software |
| Fiji Image J 2.3.0/1.53q, 1.54p | ImageJ |
| miniforge3, conda version: 24.11.3 | conda-forge |
| Python 3.9.21 | Python.org |
| Ilastik 1.4.0.post1 | Prof. Fred Hamprecht, University of Heidelberg, ilastik.org |
| Harmony 5.2 | Perkin Elmer |
| BioRender | BioRender.com |
| Leica Application Suite (LAS) 3.5.7.23225 | LEICA |
| Enersight (v1.1.0.560) | Leica Microsystems CMS GmbH |
| PyCharm 2022.2.2 | Python.org |
| Mendeley Reference Manager (v2.138.0) | mendeley.com |
| Equipment | Manufacturer/Source |
| Countess™ | Thermo Fisher Scientific |
| Leica SP8 Confocal microscope | LEICA |
| IonOptix C-Pace EM | IonOptix/CytoCypher B.V. (Europe) |
| Opera Phenix Plus | PerkinElmer, Revvity |
| Orbital Shaker | Edmund Bühler GmbH |
| NanoDrop 2000C | Thermo Fisher Scientific |
| NovaSeq X Plus system | Illumina |
| Centrifuge |  |
| Leica DMi1 Stand, with FLEXACAM C1 | Leica Microsystems CMS GmbH |
| Maestro Pro MEA platform | Axion BioSystems, USA |
| AxIS Navigator Software and Neural Metrics standalone tools | Axion BioSystems, USA |
| Stereomicroscope SZX16, with integrated DP22 camera | Olympus, Japan |
| Quantstudio 6 Flex Real-Time PCR system | Applied Biosystems |

**Table S6. Primers used for the qPCR assays**

| Gene | Primer sequence (5’-3’) |
| --- | --- |
| CHRNE *F* | GCCTGAGGATACTGTCACCATC |
| CHRNE *R* | GTCCTTGCTGTAGTTGAGTCGG |
| CHRNG *F* | CTGTCTTCCTCTTCCTTGTGGC |
| CHRNG *R* | CGACAATGAGGATGGTCACCAC |
| MYH2 *F* | GGAGGACAAAGTCAACACCCTG |
| MYH2 *R* | GCCCTTTCTAGGTCCATGCGAA |
| MYH3 *F* | CTGGAGGATGAATGCTCAGAGC |
| MYH3 *R* | CCCAGAGAGTTCCTCAGTAAGG |
| MYH4 *F* | GACAGCCAAGAAGAGGAAACTGG |
| MYH4 *R* | ACCTGCCATCTCTTCTGTGAGG |
| GAPDH *F* | GAAGGTGAAGGTCGGAGTC |
| GAPDH *R* | GAAGATGGTGATGGGATTTC |

**Supplementary Movies**

Movie S1: Representative video of day 65 WTC^mTTNGFP^ NMOs during electrical pulse stimulation training (10V, 10ms, 1Hz).

Movie S2: Representative video of day 65 KOLF NMOs during electrical pulse stimulation training (10V, 10ms, 1Hz).

Movie S3: Representative video of a day 30 NMO response to electrical pulses (10V, 10 ms, 1 Hz).

Movie S4: Representative video of a day 40 non-paced control NMO response to electrical pulses (10V, 10ms, 1 Hz).

Movie S5: Representative video of a day 40 Early-stage EPS-NMO response to electrical pulses (10V, 10ms, 1 Hz).

Movie S6: Representative video of a day 50 non-paced control NMO response to electrical pulses (10V, 10ms, 1 Hz).

Movie S7: Representative video of a day 60 Late-stage EPS-NMO response to electrical pulses (10V, 10ms, 1 Hz).

Movie S8: Representative video of a day 60 non-paced control NMO spontaneous contraction.

Movie S9: Representative video of a day 60 Early-stage EPS-NMO spontaneous contraction.

Movie S10: Representative video of a day 60 Late-stage EPS-NMO spontaneous contraction.

Movie S11: Representative video of a day 60 non-paced control NMO during electrical pulse stimulation and Curare (10nM) exposure.

Movie S12: Representative video of a day 60 non-paced control NMO spontaneous contraction.

Movie S13: Representative video of a day 60 chronic EPS-NMO (stable) spontaneous contraction.

Movie S14: Representative video of a day 60 chronic EPS-NMO (increasing) spontaneous contraction.

Movie S15: Representative video of a day 75 non-paced control NMO spontaneous contraction.

Movie S16: Representative video of a day 75 chronic EPS-NMO (stable) spontaneous contraction.

Movie S17: Representative video of a day 75 chronic EPS-NMO (increasing) spontaneous contraction.

Movie S18: Representative video of a day 60 non-paced control KOLF NMO spontaneous contraction.

Movie S19: Representative video of a day 60 chronic KOLF EPS-NMO (stable) spontaneous contraction.

Movie S20: Representative video of a day 60 non-paced control calcium activity in response to glutamate (25μM) stimulation.

Movie S21: Representative video of a day 60 chronic EPS-NMO (stable) calcium activity in response to glutamate (25μM) stimulation.

Movie S22: Representative video of a day 60 chronic EPS-NMO (increasing) calcium activity in response to glutamate (25μM) stimulation.

Movie S23: Representative video of a day 60 non-paced control NMO contraction in response to glutamate (25μM).

Movie S24: Representative video of a day 60 chronic EPS-NMO (stable) contraction in response to glutamate (25μM).

Movie S25: Representative video of a day 60 chronic EPS-NMO (increasing) contraction in response to glutamate (25μM).

Movie S26: Representative video of a day 60 non-paced control NMO during electrical stimulation for mechanobiological characterization.

Movie S27: Representative video of a day 60 chronic EPS-NMO (stable) during electrical stimulation for mechanobiological characterization.

Movie S28: Representative video of a day 60 chronic EPS-NMO (increasing) during electrical stimulation for mechanobiological characterization.

**References for Supporting Information.**

[1]	Cell Line Catalog - ALLEN CELL EXPLORER, https://www.allencell.org/cell-catalog.html .

[2]	JIPSC001000 | The Jackson Laboratory, https://www.jax.org/jax-mice-and-services/ipsc/cells-collection/JIPSC001000#.

[3]	Babraham Bioinformatics - FastQC A Quality Control tool for High Throughput Sequence Data, https://www.bioinformatics.babraham.ac.uk/projects/fastqc/ .

[4]	P. Ewels, M. Magnusson, S. Lundin, M. Käller, *Bioinformatics* **2016**, *32*, 3047.

[5]	D. Kim, J. M. Paggi, C. Park, C. Bennett, S. L. Salzberg, *Nat Biotechnol* **2019**, *37*, 907.

[6]	H. Li, B. Handsaker, A. Wysoker, T. Fennell, J. Ruan, N. Homer, G. Marth, G. Abecasis, R. Durbin, *Bioinformatics* **2009**, *25*, 2078.

[7]	Y. Liao, G. K. Smyth, W. Shi, *Bioinformatics* **2014**, *30*, 923.

[8]	M. I. Love, W. Huber, S. Anders, *Genome Biol* **2014**, *15*, 1.

[9]	G. Korotkevich, V. Sukhov, N. Budin, B. Shpak, M. N. Artyomov, A. Sergushichev, *bioRxiv* **2021**, 060012.
